# Geospatial assessment of habitat degradation and climate impacts on migratory crane habitat in Pakistan’s Wetland ecosystems

**DOI:** 10.1038/s41598-026-41758-y

**Published:** 2026-03-30

**Authors:** Muhammad Suliman, Lv Hongxue, Faisal Khalid, Wu Qingming, Sun Xueying, Majid Hussain, Zou Hongfei, Tariq Ahmad, Inam Ullah, Sami Ullah

**Affiliations:** 1 College of Wildlife and Protected Area, Northeast Forestry University, No. 26, Hexing Road, Harbin, 150040 China; 2 Department of Forestry, Shaheed Benazir Bhutto University, Khyber Pakhtunkhwa, Sheringal, 18000 Pakistan; 3 GIS and Space Applications in Geosciences Lab (GSAG-L), Institute of Space Technology, National Center of GIS and Space Application (NCGSA), Islamabad, 44000 Punjab Pakistan; 4 Department of Forestry and Wildlife Management, The University of Haripur, Haripur City, 22620 KP Pakistan; 5 Institute of Biological Sciences, Gomal University, Dera Ismail Khan, 29220 Pakistan

**Keywords:** Climate change, Migratory cranes, Habitat degradation, Flyway, Pakistan, Ecology, Ecology, Environmental sciences

## Abstract

Migratory cranes are ecologically significant avian species that depend on dynamic wetland ecosystems across their flyways. However, their habitats are increasingly threatened by anthropogenic pressures and environmental change. This study investigates the spatial and temporal dynamics of habitat suitability for the Demoiselle Crane (*Anthropoides virgo*) and Eurasian Crane (*Grus grus*) in Pakistan using multi-decadal geospatial datasets and remote sensing techniques. Supervised classification of Landsat imagery revealed significant land use/land cover (LULC) changes from 1994 to 2024, including a sharp increase in built-up areas (+ 22.4%) and a notable decrease in vegetation cover (– 4.2%), indicating intensifying habitat fragmentation and ecological stress. Key vegetation and water indices NDVI, NDWI, MNDWI, and LSWI were analyzed to evaluate ecological conditions relevant to crane habitats. Annual NDVI time-series trends indicated vegetation degradation in the early 2000s, followed by recovery after 2014 due to large-scale afforestation initiatives. Surface water dynamics, derived from the Joint Research Centre Global Surface Water dataset, showed fluctuations in water occurrence, seasonality, and recurrence factors critical for crane roosting and foraging. Climatic analysis using NASA POWER data revealed rising temperatures and variable precipitation patterns, further affecting wetland health and habitat suitability. Land Surface Flow (LSF) mapping identified critical migratory flyways along the Kurram River and Lora Nala, reinforcing the ecological importance of this corridor. This research highlights the value of geospatial tools in monitoring migratory bird habitats and underscores the urgency of integrating spatial data into national conservation policies. By identifying priority conservation zones and tracking habitat change, this study offers critical insights for the sustainable management of Pakistan’s wetlands and the protection of migratory crane populations along the Flyway.

## Introduction

Biodiversity underpins the sustainability of ecosystems and the human societies that depend on them^1^. However, rapid socioeconomic development, unsustainable land use, overexploitation of natural resources, and climate change have led to widespread ecological degradation^2^. These pressures have become the leading drivers of habitat loss, fragmentation, and degradation, contributing to the global biodiversity crisis^3^. Alarmingly, the current rate of species extinction is estimated to be 1,000 times higher than the natural background rate^4^, despite international efforts such as expanding protected areas and formulating biodiversity-focused policies^5^. Conventional habitat assessment methods are often limited in their spatial and temporal coverage^6^. However, advancements in geospatial technologies, including remote sensing (RS), geographic information systems (GIS), and global positioning systems (GPS), combined with machine learning, now enable large-scale monitoring of habitat quality and species distribution^7^. These tools are particularly valuable in tracking migratory bird responses to environmental change^8^. Cranes, with an evolutionary lineage spanning over 60 million years, are ecologically and culturally significant species^9^. The shortage of food, water, shelter, and rain causes the Demoiselle crane to move periodically. About 8,000 Demoiselle and 4,000 Eurasian cranes migrate to Bunnu, Lakki Marawat, and neighbouring tribal areas annually^10^. Between the autumn of 2008 and the spring of 2009, more than 7,000 cranes passed through the Lakki and Bannu Districts of Khyber Pakhtunkhwa^11^.

Four of the world’s 15 crane species, including the Demoiselle (*Anthropoides virgo*), Eurasian (*Grus grus*), Siberian, and Sarus Cranes, use the Indus Flyway, a key migratory route passing through the Himalayas, Karakoram, and Hindu Kush into Pakistan^12^. This region’s wetlands, including estuarine and inland systems, are among the planet’s most productive ecosystems, offering vital services such as shoreline protection, climate regulation, and critical stopover and wintering grounds for migratory birds^13^. The Demoiselle Crane, the smallest crane species, is recognized by its black neck and white ear tufts and is typically found in grasslands, wetlands, and agricultural areas, often roosting on mudflats^14^. In contrast, the larger Eurasian Crane prefers shallow marshes and wet meadows. Both species pass through Pakistan’s Sindh, Punjab, and Khyber Pakhtunkhwa (KP) provinces during winter migration^12^. Despite being listed as “Least Concern” by the IUCN (BirdLife International, 2019), both species face growing local threats^15^. In KP, cultural hunting reportedly impacts up to 15% of migratory populations annually^16^. Captive breeding practices, particularly in Bannu and Lakki Marwat, where around 8,000 Demoiselle Cranes are held, pose additional conservation concerns^17^. These include poor post-release survival and reduced genetic diversity. Habitat loss, illegal hunting, wetland encroachment, increasing human population pressures, disease transmission, urbanization, particularly tourism-related development in wetlands and weak enforcement of conservation laws further endanger crane populations^18^. Land use/land cover (LULC) changes driven by agriculture, urbanization, and infrastructure development have significantly degraded crane habitats^19,20^.

These pressures are compounded by climate change, with global temperatures now over 1.5 °C above mid-20th century levels^20^. Climate-induced range shifts and increased extinction risks threaten sensitive species like cranes^21^. According to Urbina-Cardona, Angulo^22^ habitat loss now directly affects more than 85% of vulnerable birds, mammals, and amphibians, and the combined consequences of land use and climate change increase ecosystem vulnerability. According to Wetlands International (2012), 38% of global crane populations are declining, with South Asia identified as a high-risk zone due to its dense human population, fragmented wetlands, and weak conservation capacity^23^. Migratory species like cranes are highly sensitive to temporal and spatial variations in habitat quality^24^. Understanding their responses to environmental changes across scales is essential for effective conservation planning, which includes identifying critical habitats, restoring ecological connectivity, and mitigating human-wildlife conflict^25^. Unlike earlier studies that are based mainly on LULC and vegetation water indices for assessing migratory bird habitats, this study offers a direct multi-decadal evaluation using several novel geospatial data sets and analytical techniques to the Indus Flyway. We begin by integrating Landsat-based land use/land cover metrics spanning 30 years with occurrence, seasonality and recurrence of Global Surface Water (GSW) layers in order to capture the hydrological regimes that directly affect crane roosting and foraging conditions a method seldom used in South Asian crane ecology. Secondly, by employing LSF modelling a novel spatial approach to identify likely migratory corridors along the Kurram River and Lora Nala is introduced that provides new insights on corridor connectivity in the Indus Basin. Third, this study establishes the relation of long-term NDVI trends with the time when large-scale afforestation and wetland restoration programs initiated in Pakistan process, and provide empirical evidence that national vegetation recovery program can support an ecological buffering capacity for crane habitats. By combining these integrated methodologies, the geospatial framework could readily be extended to other cranes that are widely distributed across the region and whose habitats are threatened or currently being affected by human activity and in response to changing hydro climatic conditions. In this context, geospatial tools offer a powerful framework for evaluating habitat suitability, detecting land cover changes, modelling species dispersal and other environmental changes and their effect^27,28^. This study employs GIS and remote sensing techniques to evaluate the spatial and temporal dynamics of Demoiselle (*Anthropoides virgo*) and Eurasian Crane (Grus grus) habitats in Pakistan. By analyzing land use/land cover changes, vegetation and water indices, and surface water dynamics over three decades (1994–2024), it investigates how environmental changes driven by urbanization, climate variability, and wetland degradation have affected key roosting and foraging areas. The objective is to generate geospatial insights that support evidence-based conservation planning, inform policy development, and promote the sustainable management of critical crane habitats and migratory corridors in Pakistan.

## Methodology

### Study area

**Fig. 1:**
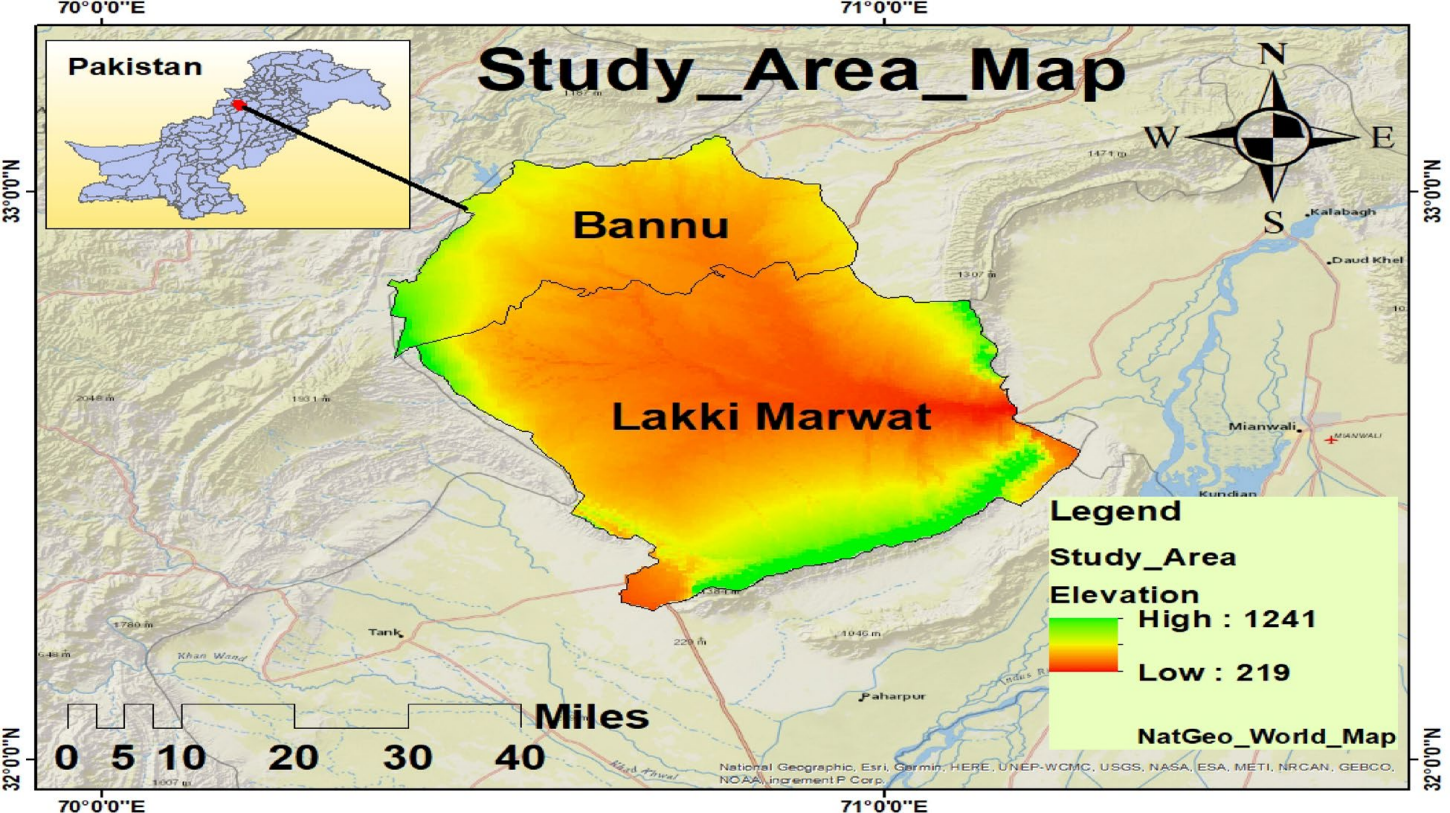
Study Area Map.

The study was carried out in the Bannu and Lakki Marwat districts of Khyber Pakhtunkhwa (KP), Pakistan’s province (Fig. 1). Both are located in a hot, semi-arid temperature zone and are connected to the Indus Flyway, which is a key crane migration path^28^. The 1,227 km² Bannu District is roughly 190 km south of Peshawar and is situated between latitudes 32°16′N and 33°5′N and longitudes 70°23′E and 71°16′E^29^. The Kurram and Gambila (Tochi) Rivers drain it, creating a basin that sustains seasonal wetlands that are essential to migratory birds^28^. With altitudes between 200 and 300 m, it is located close to 32.161°N, 70.191°E and is bounded by Isakhel (Punjab), Waziristan, Bannu, and Karak. With the Kurram and Tochi Rivers flowing through it and joining the Indus River, the district is made up of flat plains and outlying hills. Hot, dry summers (35–48 °C), mild winters (4–27 °C), little precipitation (290–350 mm), and frequent dust storms in May–June are characteristics of the climate. Because of their wetlands, croplands, and riverine ecosystems, both districts are important migratory crane stopping and wintering habitats, particularly for the Demoiselle Crane and Eurasian Crane^18^.

### Data types and source

**Fig. 2:**
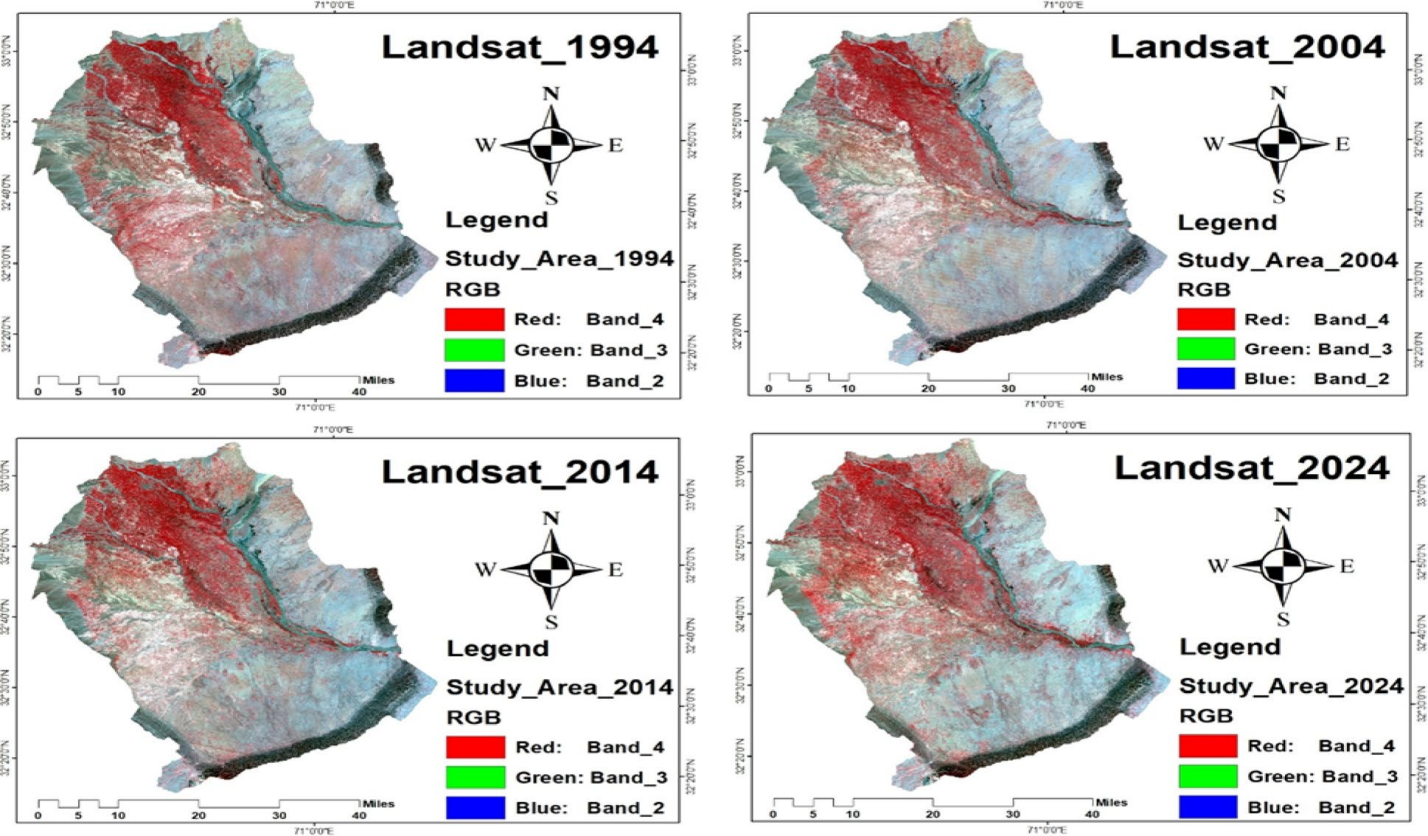
NASA POWER database.

**Fig. 3:**
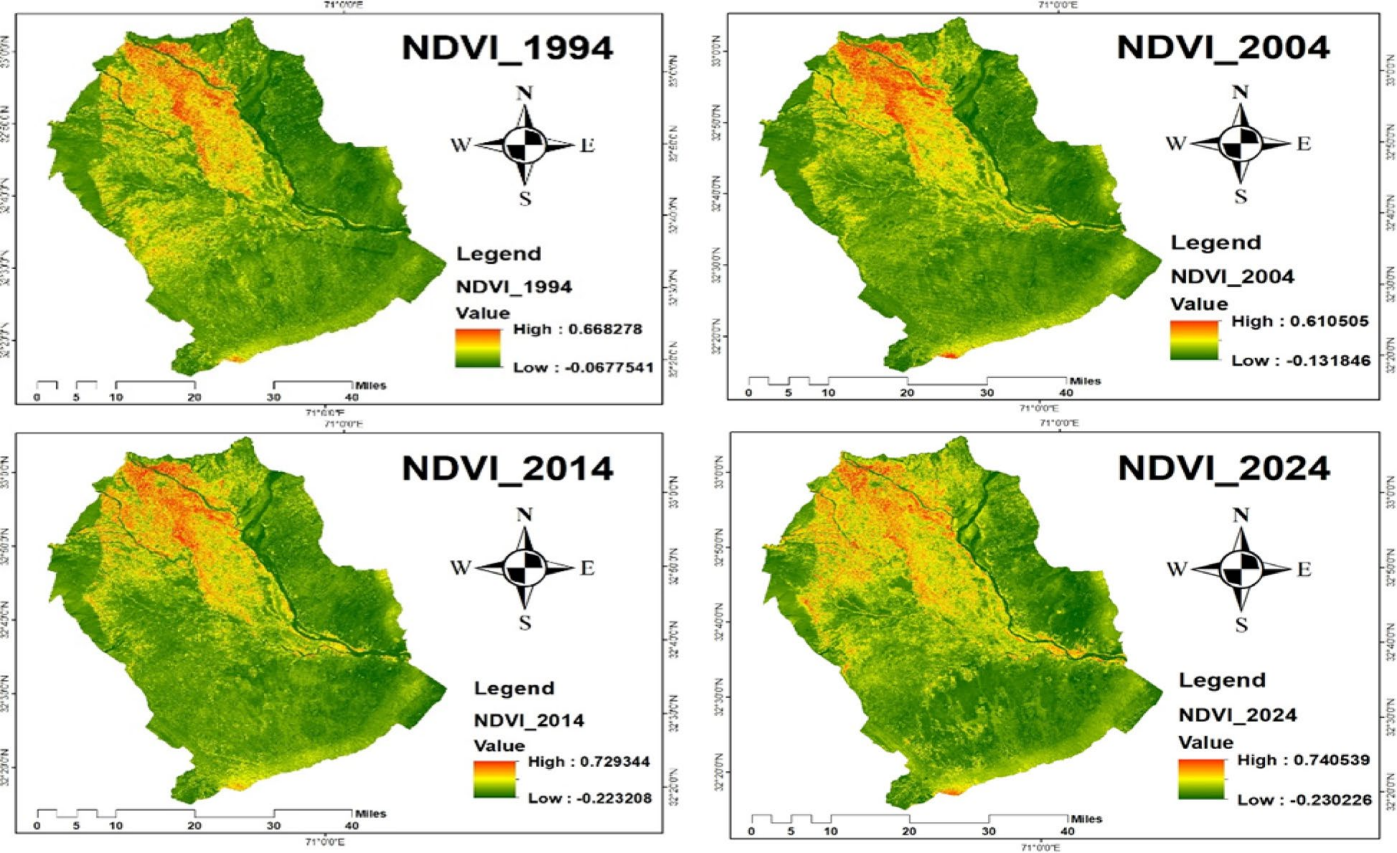
The land surface flow in the study area.

Landsat satellite images with a spatial resolution of 30 m were acquired for the years 1994 to 2024 from the U.S. Geological Survey (USGS) [https://www.usgs.gov/] and downloaded via Google Earth Engine (GEE) for further analysis. With the accumulation of large volume satellite images and the advancement of cloud computing technology, Google Earth Engine (GEE), a cloud computing platform specialized in processing geospatial data, has been increasingly applied for monitoring large-scale land cover/land use change^31,32^. To ensure high-quality data and minimize cloud interference, scenes with cloud cover less than 10% were selected. The imagery was obtained from Landsat Collection 2 Level-2 products, specifically: Landsat 4–5 TM C2 L2 for 1994, Landsat 7 ETM+ C2 L2 for 2004 and Landsat 8–9 OLI/TIRS C2 L2 for 2014 and 2024. Detailed metadata on acquisition dates, image dimensions, and sensor types is provided in Table 1. Daily temperature and precipitation data (1984–2024) were retrieved from the NASA (National Aeronautics and Space Administration) Langley Research Center (LaRC) Prediction of Worldwide Energy Resources (POWER) project funded through the NASA Earth Science/Applied Science Program [https://power.larc.nasa.gov/data-access-viewer/] as shown in Fig. 2^32^. Additionally, Global Surface Water datasets (March 1984 to December 2021) were downloaded from the Global Surface Water Data Access Portal [https://global-surface-water.appspot.com/download]^33^. These datasets, including occurrence, change, seasonality, recurrence, transitions, and maximum extent, are freely available and provided as tiled GeoTIFF files at a 10° × 10° spatial extent, as depicted in Fig. 3. Users can select and download individual datasets by clicking on specific tiles from the interactive global map interface.

**Table 1:** Metadata of Landsat imagery used in the study.

Year	Satellite/Sensor	Product Level	Cloud Cover (%)	Sensor Resolution
1994	Landsat 4–5 TM	C2 Level-2	< 10%	30 m (multispectral), 120 m (thermal)
2004	Landsat 7 ETM+	C2 Level-2	< 10%	30 m (multispectral), 15 m (panchromatic)
2014	Landsat 8 OLI/TIRS, Landsat 9 OLI-2/TIRS-2	C2 Level-2	< 10%	30 m (OLI), 100 m (TIRS), 15 m (pan)
2024	Landsat 8 OLI/TIRS, Landsat 9 OLI-2/TIRS-2	C2 Level-2	< 10%	30 m (OLI-2), 100 m (TIRS-2), 15 m (pan)

### Data pre-processing

**Fig. 4:**
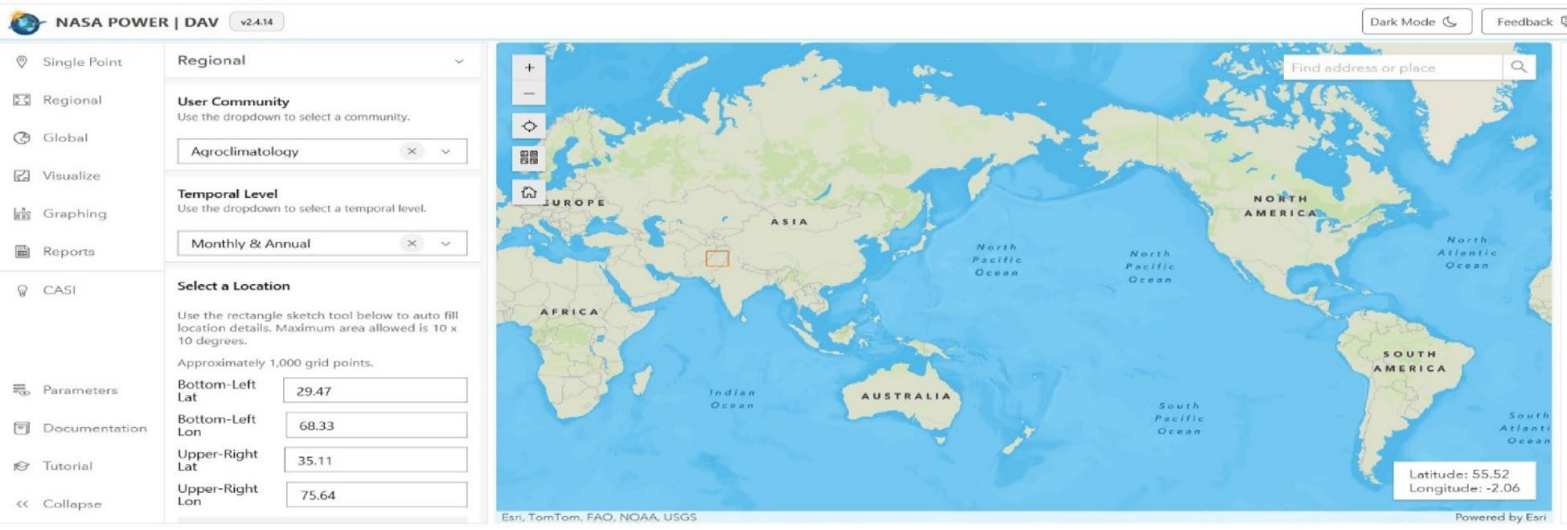
Landsat satellite images for the years 1994, 2004, 2014, and 2024.

Landsat composite images (1994–2024), as shown in Fig. 4, were generated using Google Earth Engine (GEE). A shapefile representing the study area (Bannu and Lakki Marwat districts) was uploaded to GEE and used to clip Landsat imagery to the defined extent. Cloud-free composite images were created by applying filters (cloud cover < 10%) and using the median reducer to generate annual or seasonal composites from Landsat Collection 2 Level-2 Surface Reflectance data. The composite images were then exported from GEE and processed in ArcGIS. Using the Layer Stacking tool, the spectral bands were organized for visualization and analysis. Although the data were already radiometrically and geometrically corrected by USGS, further geometric alignment was ensured using the Resampling tool to maintain spatial consistency across years. The final study area was delineated using the same shape file, and each composite was clipped using the Extract by Mask tool in ArcGIS to restrict analysis to the defined region.

### Ecological habitat criteria for cranes

To enhance the ecological validity of the geospatial analysis, habitat suitability factors for Demoiselle and Eurasian cranes were identified using published ecological requirements. Both Demoiselle and Eurasian cranes are known to prefer open vegetation, shallow water areas (< 30 cm), low disturbance habitats, and extensive roosting sites in proximity to agricultural areas and seasonal wetlands. In this study, higher NDVI values were considered to represent suitable vegetation, while NDWI, MNDWI, and LSWI values were used to determine shallow water availability, which is important for roosting and foraging. LULC categories were associated with ecological processes, where croplands and wetlands were considered foraging grounds, riverine areas were considered migratory stopovers, and urban areas represented disturbed areas. Because there were no data on species presence at a fine scale, a proxy-based suitability assessment was used, which is a limitation.

### Land use land cover (LULC) classification and change detection

A supervised classification approach was used to map Land Use Land Cover (LULC) across the study area, following the methodology of Ullah, Ullah^34^. Landsat satellite images (1994–2024), acquired via Google Earth Engine and processed in ArcGIS 10.5, were first subjected to radiometric and geometric corrections, including atmospheric correction^36,37^, to ensure accurate temporal comparisons. Multispectral band stacking and image resampling were performed to enhance the quality of classification. The LULC classification was conducted using multi-temporal datasets to identify and quantify land cover changes between years. Five major LULC categories were defined: Barren Land, Built-up Area, Crop Land, Trees, and Water Body. The classification of land use/land cover was done using the Maximum Likelihood (ML) supervised classification technique, which was chosen based on its ability to handle multispectral Landsat data effectively. The training samples were chosen using a combination of high-resolution Google Earth images, field validation, and existing land cover maps. For each LULC category, 50 to 60 polygons were delineated, representing a range of ecological conditions and spatial distribution, resulting in 250 to 300 training samples per year. Change detection analysis was conducted on a pixel-by-pixel basis to calculate the rate of change (hectares/year) across different land cover classes. Cross-tabulation analysis using the Tabulate Area tool in ArcGIS 10.5 was performed to examine the quantitative conversions among LULC categories^38–40^. Classification accuracy was assessed through 100 ground-truth points based on visual interpretation of high-resolution imagery from Google Earth. Coordinates for validation were referenced from both direct observation and literature. Change detection between 1994 and 2024 was further performed by subtracting pixel values of classified images, with threshold values applied to highlight significant transitions. A final change detection map was generated, and post-classification comparison methods were considered to improve the robustness of the results.

### Google earth interpretations

In this study, Google Earth was utilized to access historical high-resolution imagery to support land use and land cover (LULC) change detection. The time slider tool in Google Earth allowed for visual inspection of imagery from the years 1984, 1994, 2004, and 2024, enabling decadal comparisons. These images were carefully examined to identify major transitions in land cover types, such as the expansion of built-up areas, reduction in vegetation cover, and changes in water bodies. This visual assessment served as a valuable supplementary method to validate the classification outputs and helped enhance the interpretation of spatial and temporal landscape changes within the crane habitat areas.

### Accuracy assessment using confusion matrix and google earth validation

To ensure the reliability of the land use/land cover (LULC) classification, an accuracy assessment was performed using the confusion matrix approach in ArcGIS 10.5^41,42^. The accuracy of classification was evaluated independently for 1994, 2004, 2014, and 2024 on ≥ 100 randomly validation points per year. The confusion matrix, Overall Accuracy (OA), User’s Accuracy (UA), Producer’s Accuracy (PA), and Kappa statistic were calculated for each year. A combined accuracy test is also presented for comparison. A total of 100 random reference points were generated and visually verified using high-resolution imagery from Google Earth. The classified land cover types were compared against these ground-truth points to calculate standard classification accuracy metrics (Table 2). The results indicate an Overall Accuracy of 96%, demonstrating a high level of agreement between the classified map and the reference data. The Kappa coefficient was 0.93, suggesting excellent classification performance beyond random chance (Table 3). Class-wise User’s Accuracy ranged from 75% (Trees) to 100% (Built-up Area, Crop Land, and Trees), while Producer’s Accuracy ranged from 75% (Trees) to 100% (Crop Land). This assessment confirms the robustness of the LULC classification and validates the reliability of the derived land cover maps for further spatial analysis in this study.

**Table 2:** Confusion Matrix for LULC Classification Accuracy Assessment (1994–2024).

ClassValue	Water	Built_up_Area	Barren_Land	Crop_Land	Trees	Total	U_Accuracy	Kappa
Water	5.00	0.00	0.00	0.00	0.00	5.00	1.00	0.00
Built_up_Area	0.00	17.00	0.00	0.00	0.00	17.00	1.00	0.00
Barren_Land	1.00	2.00	56.00	0.00	1.00	60.00	0.93	0.00
Crop_Land	0.00	0.00	0.00	15.00	0.00	15.00	1.00	0.00
Trees	0.00	0.00	0.00	0.00	3.00	3.00	1.00	0.00
Total	6.00	19.00	56.00	15.00	4.00	100.00	0.00	0.00
P_Accuracy	0.83	0.89	1.00	1.00	0.75	0.00	0.96	0.00
Kappa	0.00	0.00	0.00	0.00	0.00	0.00	0.00	0.93

#### Kappa coefficient (K̂)

Measures agreement between classified and reference data while correcting for chance agreement:

$$K=\frac{\mathrm{P}\text{}\mathrm{o}-Pe}{1-Pe}$$

Where Po is the percentage of cases correctly classified (i.e., total accuracy) and Pe is the projected percentage of cases correctly classified by chance. Even though K can have a magnitude between − 1 and + 1, only positive values are often significant because negative values can be difficult to understand and indicate a level of agreement that is lower than that because of chance^43^. The greatest value of perfect agreement is + 1, and the value is 0 when the observed agreement is equal to the amount brought about by chance^43^. It is common to interpret the magnitude of the kappa coefficient in terms of a scale. Remote sensing applications have made considerable use of the interpretation scale proposed by Landis and Koch^45^.

#### Overall accuracy (OA)

Reflects how reliable the classification is for users (sensitive to commission error):

$$OA=\frac{\sum\mathrm{X}\mathrm{i}\mathrm{i}}{N}$$

X 100.

Whereas Xii is the number of correctly classified samples for class *i* (diagonal elements) and N is the total number of reference (ground truth) samples.

### Indices calculation

Three important remote sensing indices, the Normalized Difference Vegetation Index (NDVI), Normalized Difference Water Index (NDWI), and Land Surface Water Index (LSWI), were computed from Landsat imagery using ArcGIS software to evaluate the environmental factors affecting the habitat suitability of migratory crane species, particularly the Demoiselle Crane and Eurasian Crane. In order to help identify appropriate stopover and wintering sites for crane conservation planning, these indices collectively provided a thorough evaluation of the seasonal and geographical variations in habitat conditions throughout the research area^45^.

#### Normalized difference vegetation index (NDVI)

One of the most used vegetation indicators for tracking the health and amount of greenery worldwide is the NDVI^46^. Because of the existence of chlorophyll, healthy vegetation absorbs a large percentage of the visible electromagnetic spectrum, particularly blue light (0.4–0.5 μm) and red light (0.6–0.7 μm), while reflecting green light (0.5–0.6 μm)^48^. This is why vegetation appears green to the human eye. Furthermore, because of the interior structure of the leaves, healthy plant canopies show considerable reflectance in the near-infrared (NIR) range (0.7–1.3 μm). The NDVI is based on this difference between red light absorption and high NIR reflectance, and it is computed as:

$$NDVI=\frac{NIR-RED}{NIR+RED}$$

**Fig. 5:**
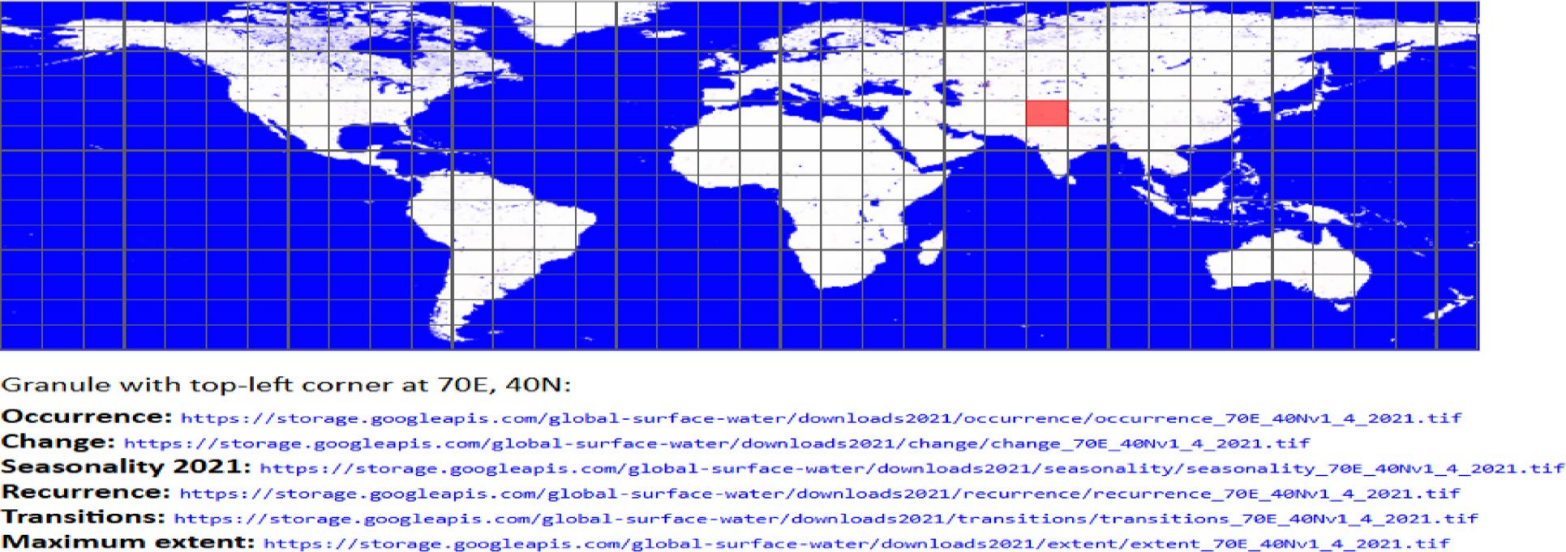
NDVI of 1994, 2004, 2014, and 2024.

Denser and healthier vegetation is indicated by higher NDVI readings, which range from − 1 to + 1. Water bodies are typically indicated by NDVI values between − 1 and 0; barren areas, such as rocks, sand, or snow, are represented by −0.1 to 0.1; shrubs, grasses, or senescing crops are indicated by 0.2 to 0.5; and dense vegetation or wooded areas are indicated by 0.6 to 1.0^48^. In order to comprehend the habitat requirements for migrating crane species, which mostly depend on green cover for foraging, breeding, and roosting during migration, the NDVI was computed (Fig. 5) using the Raster Calculator in ArcGIS 10.5.

#### Annually NDVI time-series analysis (1994–2024)

An annual NDVI time-series analysis was carried out over the study area for the years 1994 to 2024 using Landsat 5, 7, and 8 surface reflectance images obtained through the Google Earth Engine (GEE) platform^49^ in order to assess long-term vegetation dynamics pertinent to crane habitat conditions. The QA_PIXEL band bitmask was used to eliminate cloud and shadow contamination, specifically masking bits 3 (cloud) and 4 (cloud shadow)^51^. In order to convert raw digital figures into top-of-atmosphere reflectance, surface reflectance values were calibrated using sensor-specific scaling factors (multiplying by 0.0000275 and subtracting 0.2)^51^. Each image’s NDVI was determined using the conventional NDVI calculation. A continuous annual NDVI time series from 1994 to 2024 was produced by generating annual median composites for each year and extracting the mean NDVI values within the research area. To make it easier to understand inter-annual variability, the annual NDVI data were displayed in tabular style rather than as charts that visualized the trends. Understanding the seasonal foraging and roosting needs of migratory cranes requires knowledge of long-term changes in vegetation greenness and habitat quality, which Table 5 offers. The mean and median variations (1994–2024) were also calculated using ArcGIS to assess their relevance to crane habitat.

#### Trend analysis

Temporal patterns of NDVI, temperature, and precipitation were analyzed to assess the magnitude of environmental changes and their implications for crane habitat. The Mann-Kendall trend test was employed to identify the significance of monotonic trends, and Sen’s slope estimator was employed to identify the nature and magnitude of trends during the period of interest. The significance of trends was determined at a level of *p* < 0.05. The data on NDVI was analyzed from 1994 to 2024, while temperature and precipitation data were analyzed from 1984 to 2024.

#### Normalized difference water index (NDWI)

Remote sensing photography is frequently used to identify and analyze surface water bodies using the Normalized Difference Water Index (NDWI)^52^. The near-infrared (NIR) and shortwave infrared (SWIR) bands are used by NDWI, which was first created by McFeeters^53^, to highlight water-related features in a landscape. It is computed as:

$$NDWI=\frac{NIR-SWIR}{NIR+SWIR}$$

However, because both NIR and SWIR reflectance are low over open water surfaces, this variant frequently performs poorly when it comes to precisely identifying clean water bodies. In order to overcome this restriction, Xu^54^ suggested a modified NDWI that improves detection accuracy in turbid and built-up environments by using the green band rather than NIR. Particularly when it comes to clear or turbid water, water often has a low reflectance in the NIR and SWIR regions but a high reflectance in the blue (0.4–0.5 μm) and green (0.5–0.6 μm) portions of the electromagnetic spectrum. NDWI is a useful tool for monitoring habitat conditions for water-dependent species like migratory cranes, identifying wetland loss or gain, and contextualising changes in land cover, especially when combined with NDVI^55^.

#### Modified normalized difference water index

By substituting the green band for the Near-Infrared (NIR) band, the Modified Normalised Difference Water Index (MNDWI), put forth by Xu^54^, enhances the detection of water bodies and makes it more successful in differentiating water from vegetation and populated regions. The following formula is used to calculate MNDWI:

$$MNDWI=\frac{Green-SWIR}{Green+SWIR}$$

The resulting values fall between − 1 and + 1, with values above 0.5 generally denoting bodies of open water. On the other hand, vegetation shows significantly lower MNDWI values, which are frequently negative, making it possible to distinguish it from water. Low positive readings, usually between 0 and 0.2, are commonly found in built-up areas. Because of its improved spectral separation, MNDWI is especially useful for tracking water features in complex or urban landscapes. It also offers vital information about the availability of wetlands for migratory waterbirds, like cranes, which depend on surface water bodies for feeding and roosting during migration.

#### Land surface water index (LSWI)

A popular spectral index for identifying surface water and determining vegetation water stress in remote sensing applications is the Land Surface Water Index (LSWI)^57,58^. It works especially well for drought analysis, wetland mapping, and agricultural monitoring. For LSWI, the typical formula is:

$$LSWI=\frac{NIR-SWIR}{NIR+SWIR}$$

While negative readings (LSWI < 0) indicate dry soil or vegetation water stress, positive values (LSWI > 0) generally indicate high water content in plants or the presence of surface water.

### Analysis of NASA power data

The temperature and precipitation data, obtained from the NASA POWER database (Fig. 2) in NetCDF format, were analyzed using ArcGIS 10.5. The NetCDF files were first converted into raster layers using the “Make NetCDF Raster Layer” tool available in the Arc Toolbox^59^. This process generated multi-band outputs (Red, Green, Blue), which were then exported for further analysis. Precipitation data were processed using the “Cell Statistics” tool from the Spatial Analyst toolbox to compute average precipitation across the study period^59^. All raster datasets were subsequently projected using the “Project Raster” tool (Data Management Tools) to convert from GCS_WGS_1984 to WGS_1984 UTM Zone 43 N. To enhance the spatial resolution of the raster layers, each raster was converted to a point feature using the “Raster to Point” tool. This conversion assigned a specific value to each cell, stored in the “Grid Code” attribute field. The Inverse Distance Weighted (IDW) interpolation method was then applied using the Grid Code field to estimate continuous surfaces from the point data. The IDW technique interpolates values at unsampled locations based on known values from surrounding points^61^. Despite the availability of NASA POWER temperature and precipitation data in the form of gridded raster’s, IDW interpolation was used to create continuous surfaces that are spatially consistent with Landsat raster layers. The raster layers were then clipped to the study area using the “Extract by Mask” function in ArcGIS 10.5. This is helpful for combining climate data with remote sensing data without losing the original spatial and temporal patterns. After interpolation, the study area was clipped using the “Extract by Mask” tool to retain only relevant data. This entire process was performed separately for precipitation (1984–1994) and temperature (1984–1994) datasets to generate high-resolution climatic surfaces for use in ecological and habitat analyses.

### Analysis global surface water dataset

**Fig. 6:**
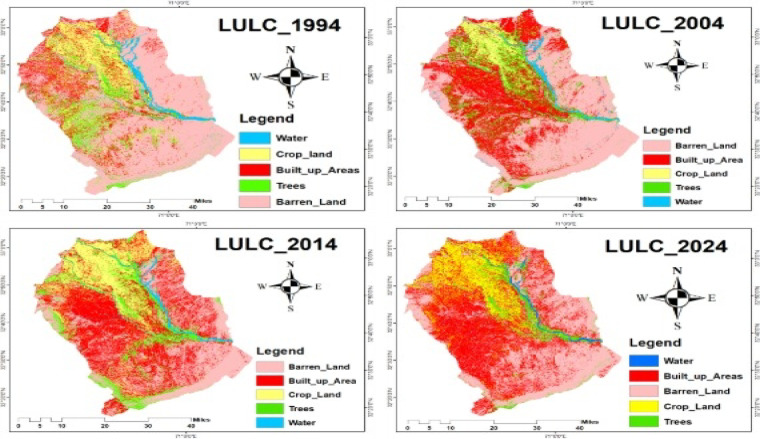
Classified Images of 1994, 2004, 2014, and 2024.

The Global Surface Water datasets (Fig. 6) developed by the European Commission Joint Research Centre (JRC) were downloaded and analyzed using ArcGIS 10.5. Upon importing, all datasets were reprojected using the “Project Raster” tool in Data Management Tools to ensure consistency with the coordinate system of the study area. The area of interest was extracted using the “Extract by Mask” tool to isolate the relevant region from the global dataset. Final map outputs were generated based on guidelines provided in the Global Surface Water - Data Users Guide (v4), which offers a comprehensive technical overview of each dataset, including purpose, description, data bands, and symbology. This documentation enabled the correct interpretation and visualization of the layers, such as occurrence, change, seasonality, recurrence, and maximum water extent. Much of the methodological background is drawn from the reference publication by Pekel, Cottam^33^, which details the remote sensing techniques and algorithms used in generating the datasets. These processed water datasets were used to assess long-term surface water dynamics and wetland conditions, providing valuable insight into seasonal habitat availability for migratory cranes.

## Results and discussion

### Results

#### LULC pattern of the study area (1994–2024) and its effect on crane habitat

The Land Use Land Cover (LULC) classification maps for the years 1994, 2004, 2014, and 2024 are shown in Fig. 6, while the corresponding area statistics for each land category are summarized in Table 3. The data were classified using a supervised classification method into five land cover classes: Barren Land, Built-up Area, Cropland, Trees, and Water Bodies, respectively. The analysis reveals significant land cover changes over the 30 years, with direct implications for crane habitat availability and quality. The most substantial decrease occurred in the Barren Land category, which declined by 47,958.0 hectares, representing a 22.6% reduction of the total area from 1994 to 2024. This suggests that formerly open or unused lands potential resting or feeding areas for migratory cranes—have been altered. In contrast, the Built-up Area showed a sharp increase of 171,108.4 hectares, accounting for a 22.4% expansion, indicating rapid urbanization and habitat fragmentation. Cropland and Water bodies also expanded by 47,545.2 hectares (3.8%) and 8,584.1 hectares (0.6%), respectively, likely driven by agricultural intensification and water management practices. Meanwhile, the Tree cover and Built-up vegetation area decreased by 11,443.9 hectares and 4.2% respectively, reflecting ongoing deforestation and land conversion pressures. These land cover transitions, particularly the loss of barren land and tree cover, may reduce suitable roosting and foraging sites for cranes, while increased urban and agricultural activities can lead to disturbance and habitat degradation. Monitoring such trends is critical for informing conservation strategies along the Indus Flyway. The overall accuracy and Kappa coefficient both indicate that the outstanding classification accuracy exceeded 90%, as presented in Table 3.

**Table 3:** Land Use Land Cover (LULC) Area Statistics and Percentage Change from 1994 to 2024 in the Study Area.

Barren Land	1994	%age	2004	%age	2014	%age	2024	%age	LULC Change	%age	Trend
	290241.3	59.6	439143.7	56.8	325753.6	46.4	242283.3	37.0	−47958.0	−22.6	↓
Built-up Areas	71174.9	14.6	191686.5	24.8	207216.8	29.5	242283.3	37.0	171108.4	22.4	↑
Cropland	65811.3	13.5	31303.7	4.0	109374.8	15.6	113356.6	17.3	47545.2	3.8	↑
Trees	46348.1	9.5	100106.3	12.9	49260.2	7.0	34904.2	5.3	−11443.9	−4.2	↓
Water	13463.1	2.8	10810.6	1.4	9878.2	1.4	22047.2	3.4	8584.1	0.6	↑
Overall Accuracy	0.96		0.95		0.94		0.96				
Kappa	0.93		0.91		0.90		0.9				

#### Natural and anthropogenic drivers of LULC change based on google earth interpretation

**Fig. 7:**
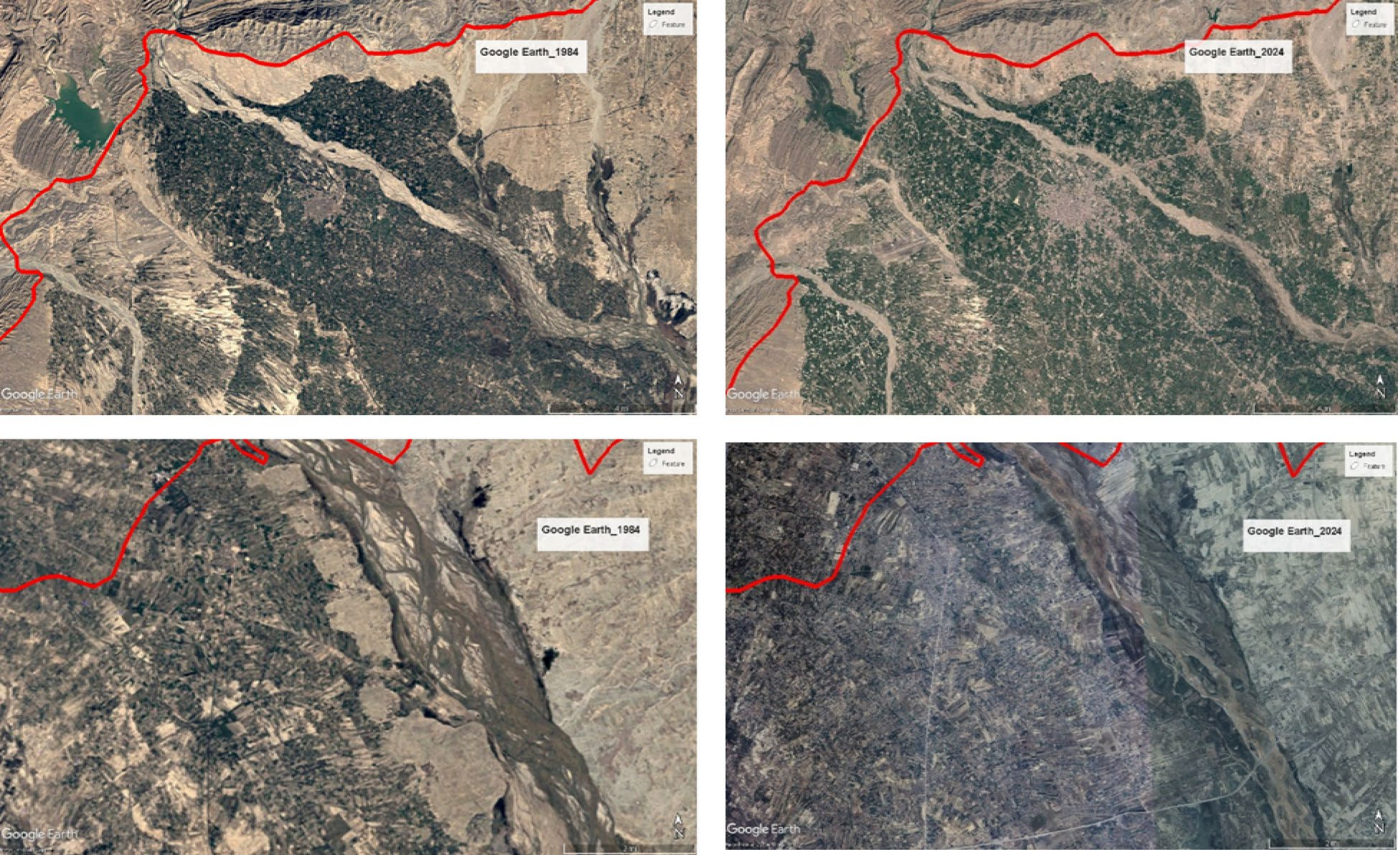
Visual Interpretation of LULC Changes (1994–2024) Using Google Earth Imagery.

The observed LULC changes in the study area are driven by both natural processes and anthropogenic activities^61^. Land cover dynamics are inherently complex, and without distinguishing the relative contributions of climate and human-induced drivers, the understanding of LULC change mechanisms remains incomplete^62^. Figure 7 illustrates changes in land cover at identical locations using historical Google Earth imagery from 1994 to 2024, clearly showing urban expansion, building construction, and other landscape alterations. This visual interpretation of temporal imagery in Google Earth effectively supports and cross-validates the satellite-based LULC classification results, highlighting the accuracy of observed patterns of change.

#### Change detection analysis from 1994 to 2024

**Fig. 8:**
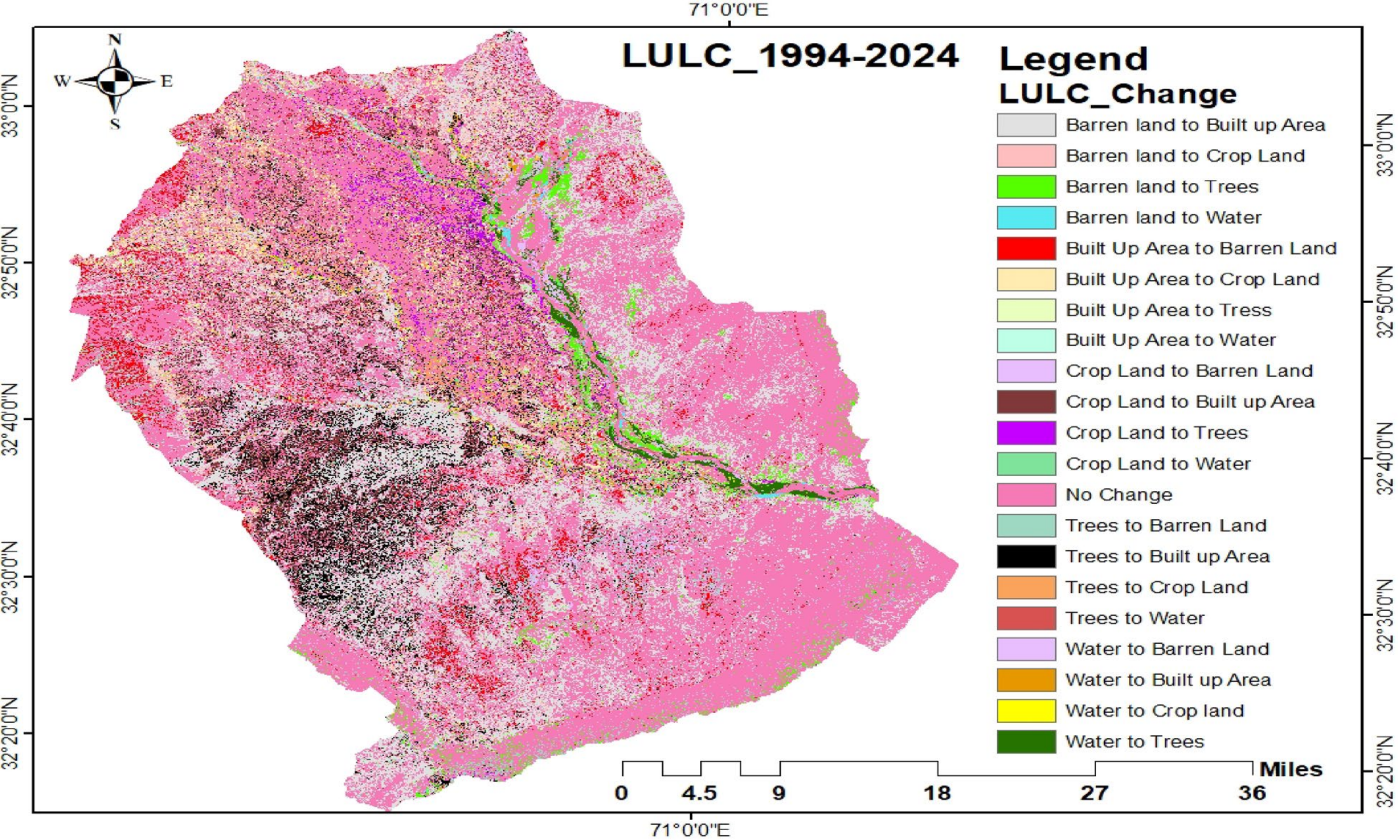
Change detection map showing LULC transitions between 1994 and 2024.

Change detection analysis is conducted not only to identify the changes that have occurred over time, but also to understand the nature, spatial distribution, and areal extent of those changes^64,65^. The analysis of LULC transitions among five major categories—Barren Land, Built-up Area, Cropland, Trees, and Water Bodies from 1994 to 2024 is summarized in Table 4; Fig. 8. Approximately 250,409 hectares (51.4% of the total area) remained unchanged over the 30-year period. The most prominent transformation was the conversion of Barren Land to Built-up Area (98,633.8 ha; 20.3%), followed by Trees to Built-up Area (21,207.5 ha; 4.4%) and Cropland to Built-up Area (21,085.3 ha; 4.3%). Some areas originally classified as Built-up reverted to Barren Land (18,290.3 ha; 3.8%) or Cropland (14,490.8 ha; 3.0%). Other transitions were relatively minor and are detailed in Table 4. These land cover changes have substantial implications for crane habitats. Rapid expansion of Built-up Area reflects population growth and urbanization, which intensify pressure on natural ecosystems through habitat fragmentation, pollution, and increased resource extraction. Such disturbances reduce available foraging and roosting sites and may contribute to long-term population declines. The conversion of open and wooded lands to urban use represents the primary pathway of habitat loss. Although these habitat patches may be smaller than the total area used by migratory cranes, they serve as critical refuges and stopover sites during migration periods.

**Table 4:** Area (ha) and Percentage of LULC Transitions Between Major Classes from 1994 to 2024.

LULC Change	Area	%age
No Change	250409.0	51.4
Barren Land to Built-up Area	98633.8	20.3
Barren Land to Crop Land	8795.0	1.8
Barren land to Trees	9371.8	1.9
Barren land to Water	1386.1	0.3
Built-Up Area to Barren Land	18290.3	3.8
Built-Up Area to Crop Land	14490.8	3.0
Built Up Area to Trees	2777.5	0.6
Built-Up Area to Water	298.8	0.1
Crop Land to Barren Land	5113.5	1.1
Crop Land to Built-up Area	21085.3	4.3
Crop Land to Trees	5282.8	1.1
Crop Land to Water	207.6	0.0
Trees to Barren Land	9853.3	2.0
Trees in Built up Area	21207.5	4.4
Trees to Crop Land	9303.1	1.9
Trees to Water	797.1	0.2
Water to Barren Land	3516.7	0.7
Water to Built-up Area	1313.5	0.3
Water to Crop land	669.5	0.1
Water to Trees	4176.8	0.9

#### Temporal NDVI, mean, and median variation (1994–2024) Indicating vegetation dynamics and their relevance to crane habitat

**Fig. 9:**
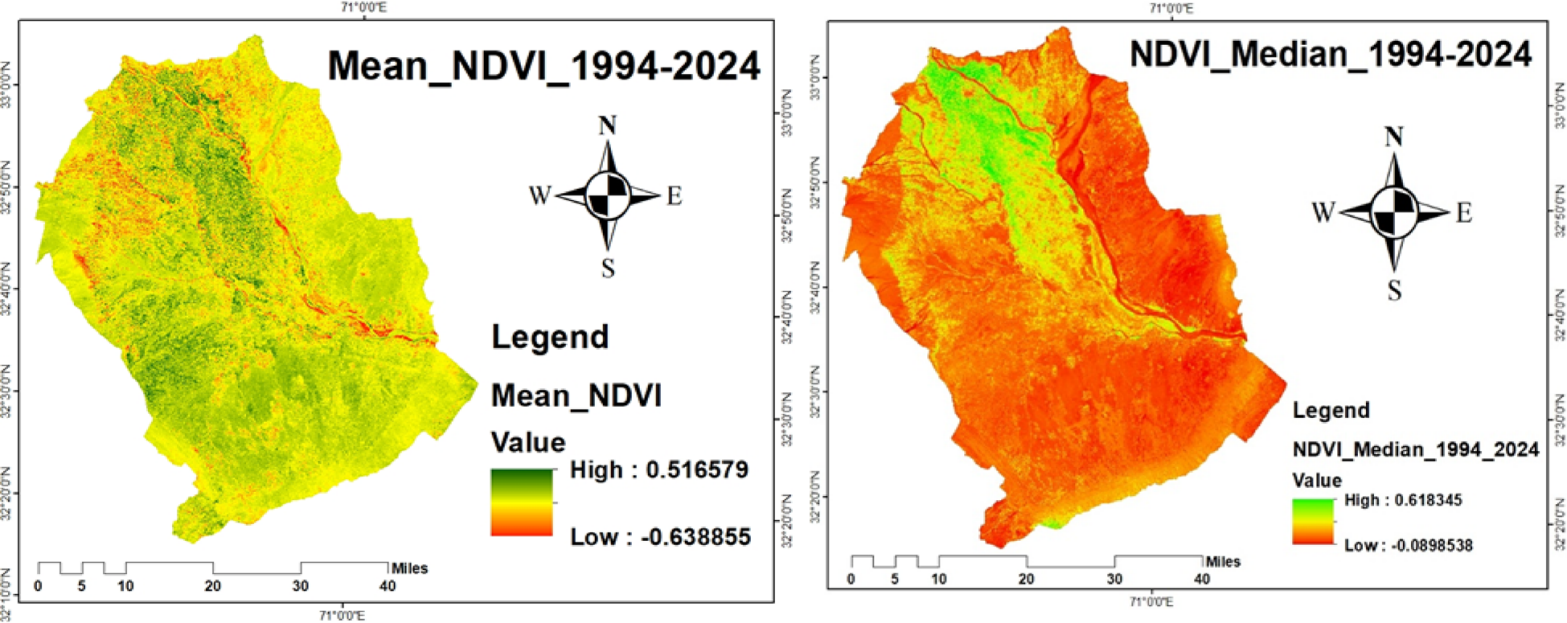
Temporal NDVI Variation from 1994 to 2024 Indicating Vegetation Dynamics.

The Normalized Difference Vegetation Index (NDVI) is considered one of the most effective and widely used indices for assessing vegetation dynamics globally^46^. In this study, NDVI was calculated for the years 1994, 2004, 2014, and 2024 to evaluate changes in vegetation cover across the study area. The NDVI values in 1994 ranged from − 0.06 to 0.67, while in 2004, the values ranged from − 0.13 to 0.61. For 2014, NDVI values extended from − 0.20 to 0.72, and in 2024, the lowest value remained − 0.20, with a maximum of 0.74, as illustrated in Fig. 5. The summary statistics of NDVI across all years indicate that the mean NDVI ranged from − 0.63 to 0.51, and the median NDVI varied between − 0.08 and 0.61, as shown in Fig. 9. These temporal changes in NDVI reflect variations in vegetation health, density, and distribution. When comparing NDVI values across the decades, the results highlight substantial shifts in vegetative cover, likely driven by urbanization, agricultural expansion, drought, and deforestation^66,67^. These NDVI dynamics are directly linked to the availability and quality of habitat for migratory crane species, such as the Demoiselle Crane and the Eurasian Crane, which depend on healthy vegetation and wetlands for foraging and roosting. A decline in vegetation cover due to LULC change reduces the suitability of stopover and wintering sites, threatening crane population sustainability. Conversely, areas with increasing NDVI may represent improved or restored habitats essential for crane survival during migration.

#### Annually NDVI time-series analysis (1994–2024)

The annual changes in NDVI values from 1994 to 2024 are summarized in Table 5. Initially, in 1994–1995, the NDVI was recorded at 0.15, followed by a steady decline up to around 2010, indicating vegetation degradation. However, a positive shift began in 2013, with NDVI increasing to 0.17 and further rising to 0.21 by 2024. A notable factor contributing to this upward trend is the launch of large-scale afforestation initiatives, including the Billion Tree Afforestation Project in Khyber Pakhtunkhwa in 2014^67,68^, and the 10 Billion Tree Tsunami Project initiated later across Pakistan^69,70^. These efforts have played a significant role in restoring vegetation, thereby improving the quality and extent of crane habitats in the region.

**Table 5:** Annual NDVI Trends (1994–2024) and the Impact of National Afforestation Programs on Crane Habitat Restoration.

Mean NDVI	Years	Mean NDVI	years
0.15	1994	0.13	2010
0.15	1995	0.15	2011
0.14	1996	0.13	2012
0.14	1997	0.17	2013
0.13	1998	0.17	2014
0.13	1999	0.18	2015
0.11	2000	0.17	2016
0.10	2001	0.16	2017
0.10	2002	0.15	2018
0.12	2003	0.18	2019
0.11	2004	0.19	2020
0.12	2005	0.16	2021
0.11	2006	0.20	2022
0.14	2007	0.21	2023
0.12	2008	0.21	2024
0.13	2009		

#### Normalized Difference Water Index (NDWI)

**Fig. 10:**
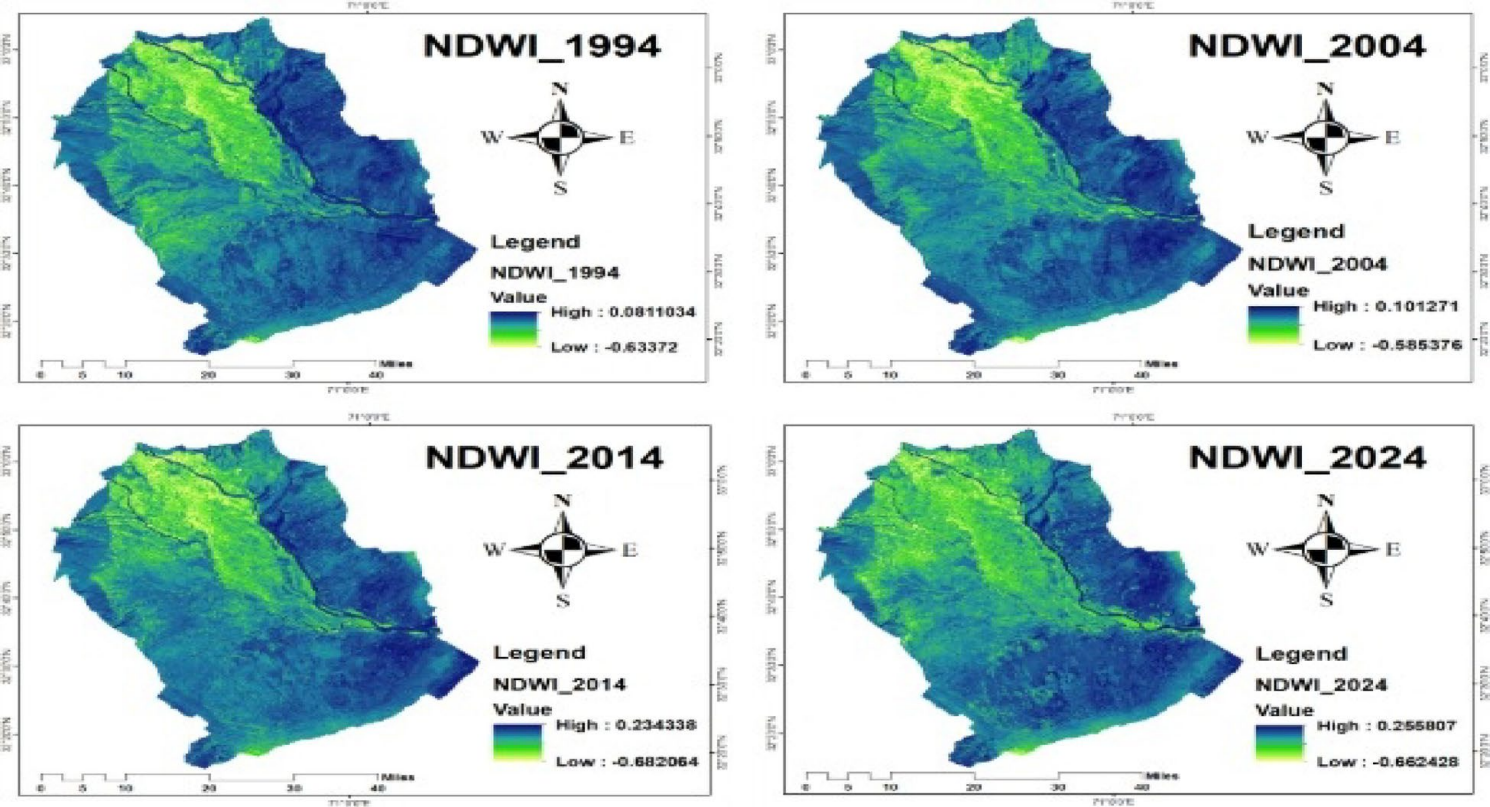
NDWI of 1994, 2004, 2014, and 2024.

The NDWI values showed temporal variation across the study area, indicating changes in surface water presence. In 1994, NDWI ranged from − 0.63 to 0.08; in 2004, from − 0.58 to 0.10; in 2014, from − 0.68 to 0.23; and in 2024, from − 0.66 to 0.25, as shown in Fig. 10. The gradual increase in NDWI values over time suggests improved surface water conditions, which are essential for maintaining suitable wetland habitats for migratory crane species.

#### Modified normalized difference water index (MNDWI)

**Fig. 11:**
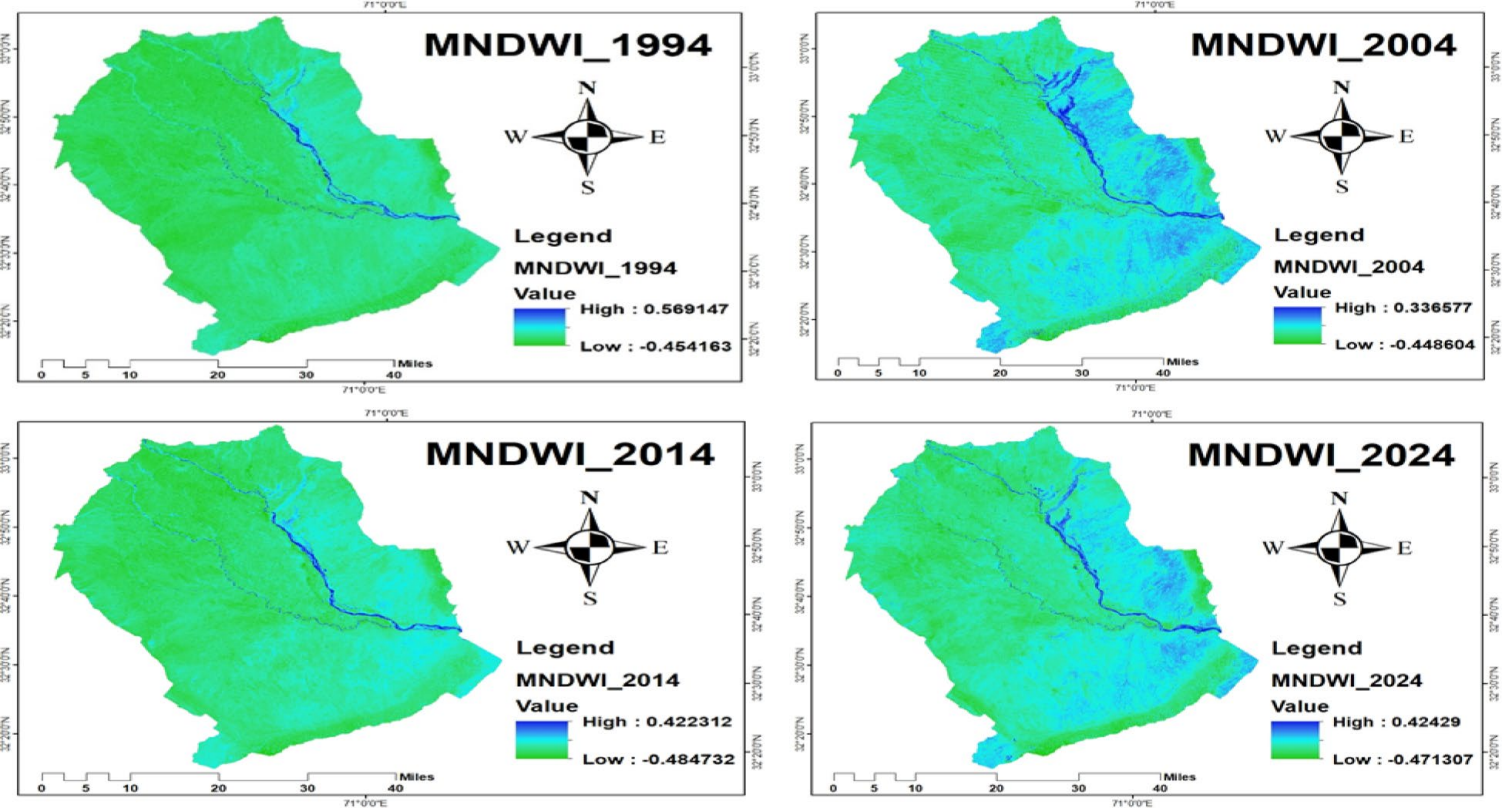
Modified normalized difference water index for the years 1994, 2004, 2014, and 2024.

The Modified Normalized Difference Water Index (MNDWI) values varied across the study years, reflecting surface water dynamics in the region. In 1994, MNDWI ranged from − 0.45 to 0.56; in 2004, from − 0.44 to 0.33; in 2014, from − 0.48 to 0.42; and in 2024, from − 0.47 to 0.42, as illustrated in Fig. 11. These values indicate fluctuations in water body extent over time, which can influence the availability and quality of wetland habitats crucial for crane foraging and roosting. Thus, monitoring surface water dynamics using indices such as MNDWI is crucial for understanding the habitat conditions of migratory cranes and for guiding effective conservation efforts in Pakistan’s wetlands.

#### Land surface water index **(LSWI)**

**Fig. 12:**
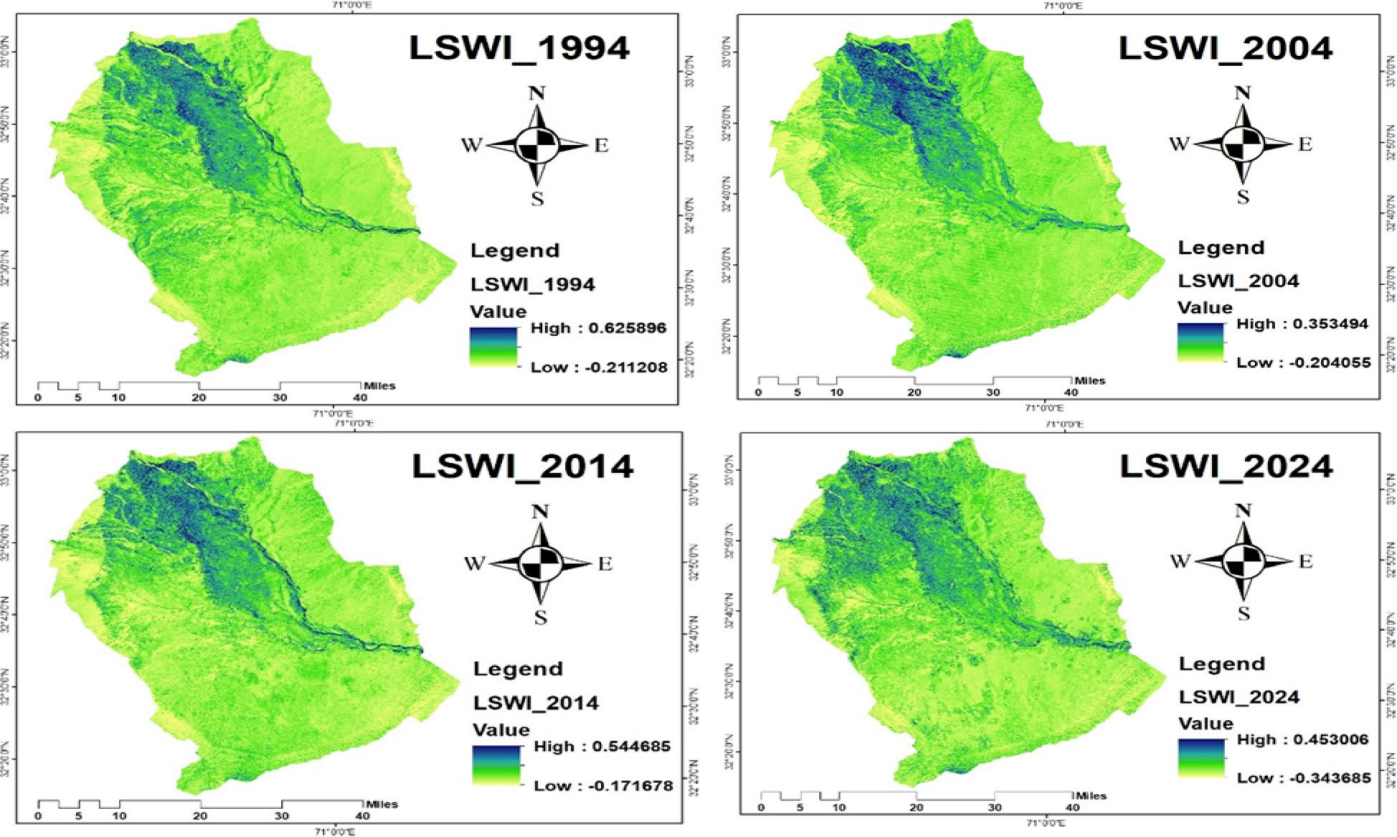
LSWI of 1994, 2004, 2014, and 2024.

The land surface water plays a significant role in crane movement, habitat suitability, and population dynamics^70^. The Land Surface Water Index (LSWI) analysis revealed notable variations over the study period, indicating fluctuations in surface moisture and vegetation water content. The results demonstrated a decline in LSWI values in 2004, followed by a slight increase in 2014, and then a decrease again in 2024, suggesting changing land surface conditions over time. Specifically, in 1994, LSWI values ranged from − 0.21 to 0.62; in 2004, from − 0.20 to 0.35; in 2014, from − 0.17 to 0.54; and in 2024, from − 0.34 to 0.45, as presented in Fig. 12. These trends reflect changing surface water and vegetation moisture levels, which directly influence the availability and quality of crane habitats.

#### Climate trends analysis and implications for crane habitat (1984–2024)

**Fig. 13:**
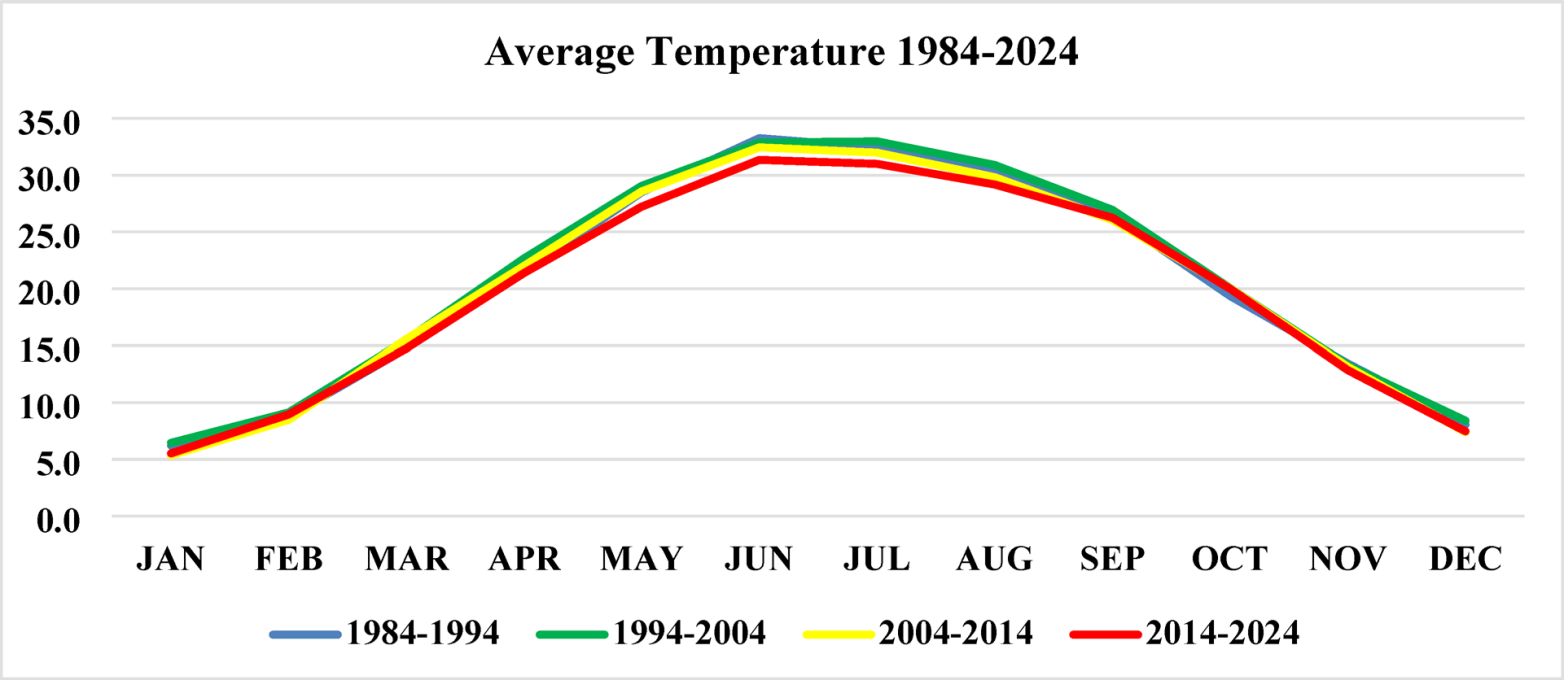
Average temperature from 1984 to 2024.

**Fig. 14:**
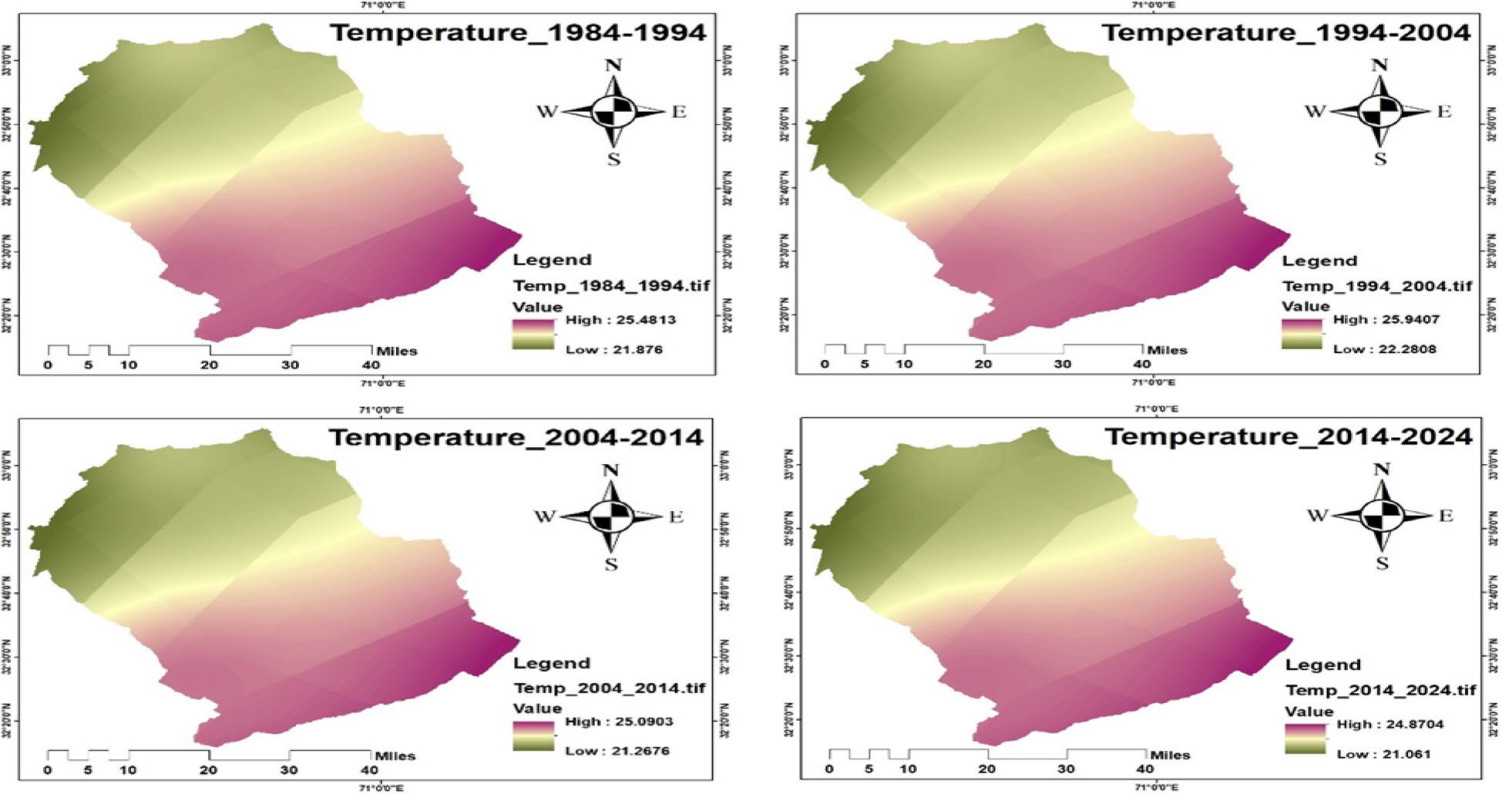
Temperature map from 1984 to 2024.

The results of NASA Power data analysis include temperature and precipitation data from 1984 to 2024, which are critical for understanding long-term environmental conditions influencing crane habitat suitability. The average temperature has shown a slight increase, especially during the summer months (June and July), as presented in Figs. 13 and 14.

Precipitation patterns also demonstrated significant variation, particularly during the monsoon season (June–September), with an increasing trend by 2024, Figs. 15 and 16. According to national climate reports, August 2013 recorded the highest average daily rainfall (21.6 mm/day) at the Sialkot station in the last 76 years. The Islamabad region had the highest total annual precipitation. These hydrological shifts can significantly influence wetland size, surface water availability, and food resources^71^, all of which are vital for crane migration and staging areas. Climate variability in temperature and precipitation is closely linked to crane ecology, influencing migration timing, habitat availability, and overall survival. Historical climate data from World Data Info for Pakistan (https://www.worlddata.info/asia/pakistan/climate.php) (Karachi, Multan, Lahore, Jhelum) indicate that June 1994 was the hottest month (34.6 °C) and January 2024 the coldest (12.2 °C). Over the last four decades, the annual mean temperature has risen by about 0.9 °C, a trend that affects wetland hydrology and vegetation essential for Demoiselle and Eurasian cranes. Changes in temperature and monsoon rainfall directly alter water bodies and wetland conditions, shaping suitable foraging and roosting habitats. Integrating climatic trends with NDVI and wetland-water data helps identify high-quality crane habitats and areas vulnerable to ecological degradation.

**Fig. 15:**
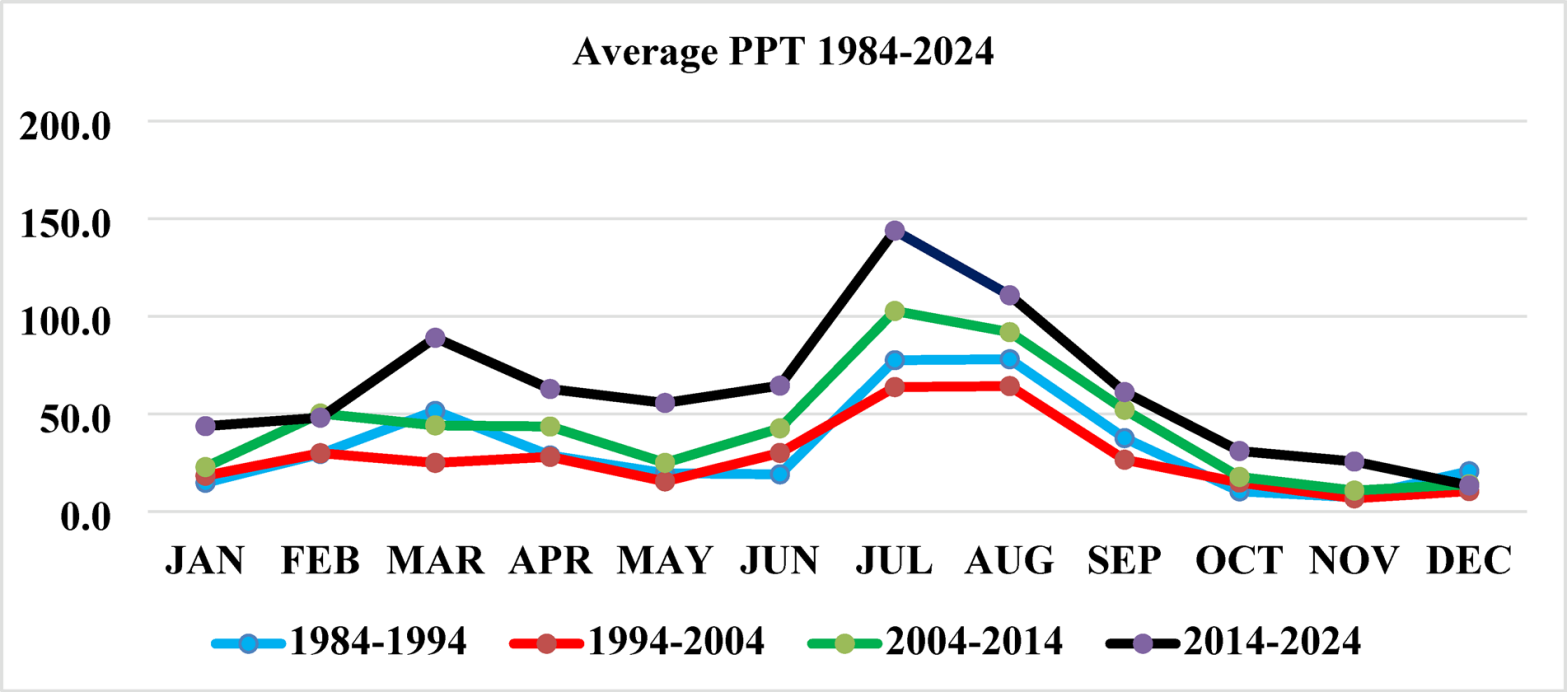
Average precipitation from 1984–2024.

**Fig. 16:**
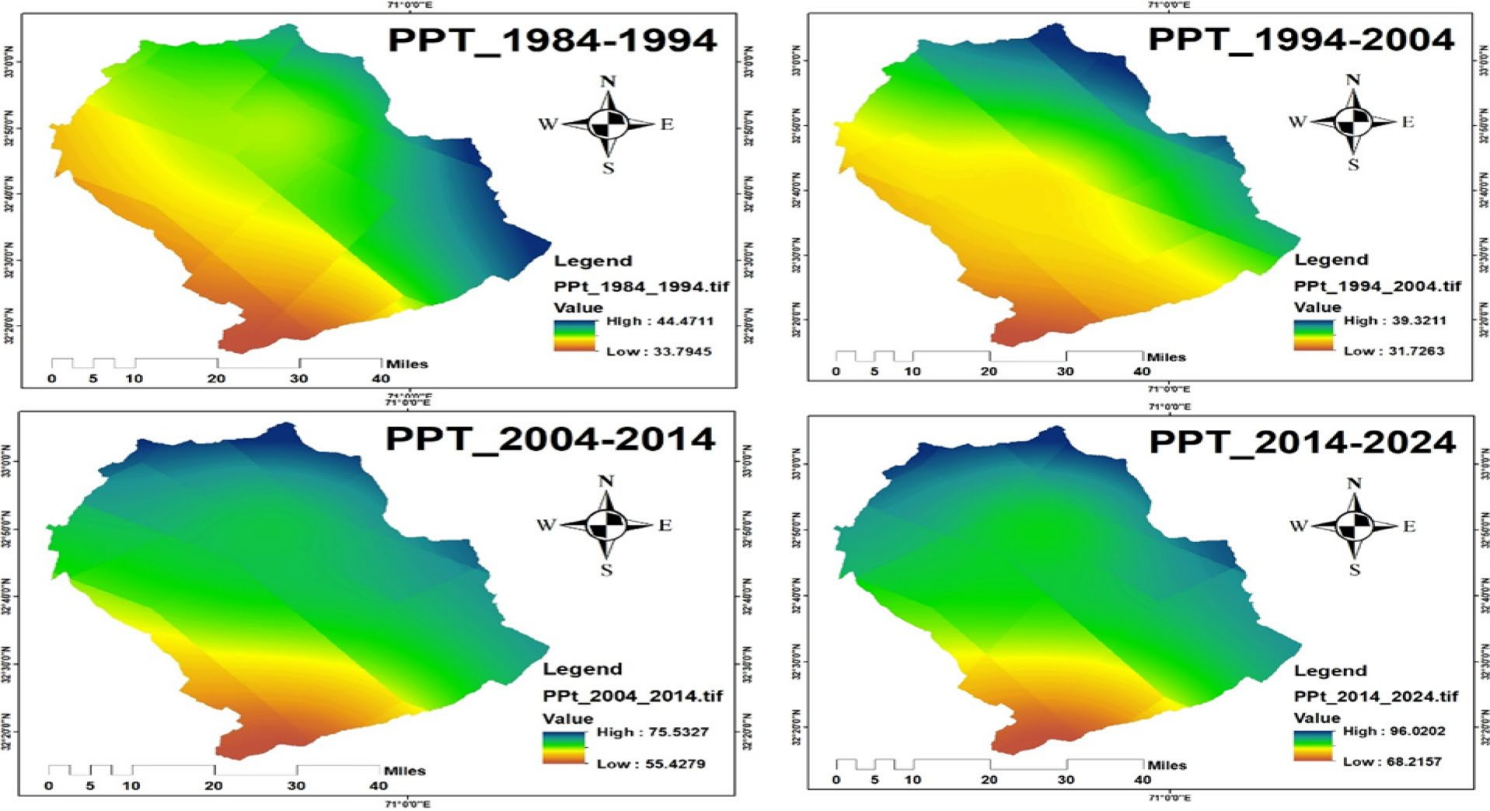
Precipitation map from 1984 to 2024.

#### Trend analysis of NDVI, temperature, and precipitation

The formal statistical analysis indicates the presence of significant trends in vegetation and climate variables that affect the crane habitats. The NDVI values indicated a decline until around 2010, followed by an increasing trend that aligns with the national afforestation program, indicating an improvement in vegetation cover that is suitable for crane foraging and roosting. Temperature trends indicate a slight increase over the last four decades (~ 0.9 °C), especially during the summer season, while the precipitation trend indicates a modest increase, especially during the monsoon season. Formal trend analysis (Table 6) reveals the following trends: NDVI = 0.0023 per year, Temperature = 0.022 °C per year, Precipitation = 0.45 mm per year. The trends suggest that the vegetation conditions and availability of water resources are improving after 2013, which are crucial for crane foraging, roosting, and stopover sites. The habitat characteristics found throughout the landscape vary spatially. Comparably high NDVI or have stable access to water indicates new suitable habitats for cranes, while areas that are primarily urban and/or have agricultural land will be degraded.

**Table 6:** Trends in NDVI, Temperature, and Precipitation (1994–2024/1984–2024).

Variable	Period	Sen’s Slope (per year)	Trend Direction	Mann–Kendall *p*-value
NDVI	1994–2024	+ 0.0023	↑	0.012
Temperature (°C)	1984–2024	+ 0.022	↑	0.034
Precipitation (mm/year)	1984–2024	+ 0.45	↑	0.041

#### Global surface water dataset and Implications for Crane Habitat (1984–2024)

**Fig. 17:**
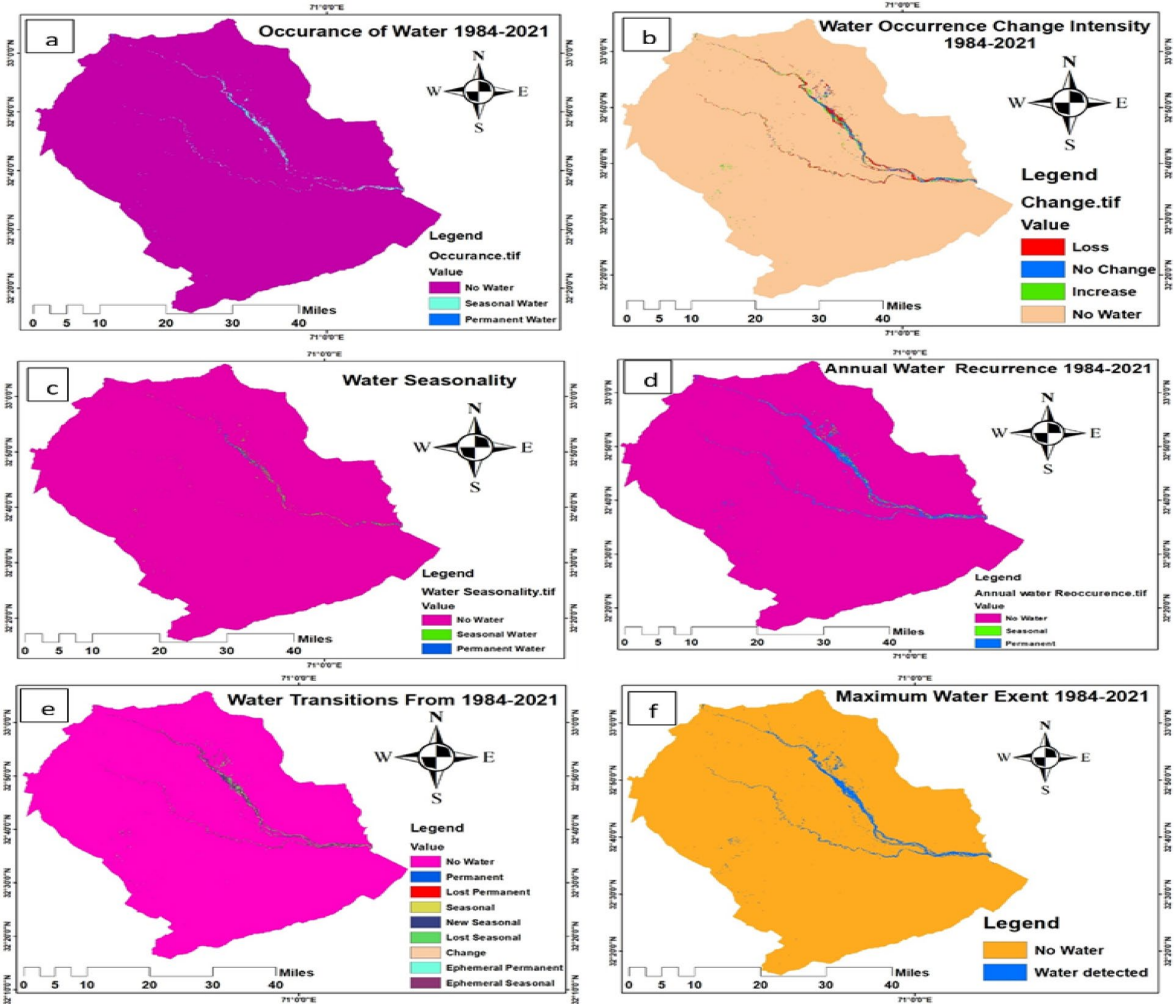
Occurrence, change, seasonality, recurrence, transection, and maximum extent of water from 1984 to 2021.

The Global Surface Water dataset (1984–2024) offers comprehensive insight into long-term hydrological patterns and their implications for crane habitat by analyzing six core indicators: occurrence, change intensity, seasonality, recurrence, transitions, and maximum extent. The Water Occurrence product (Fig. 17a) maps where surface water appeared from 1984 to 2021 by averaging monthly detections over valid observations, reducing seasonal bias. In this map, dark blue shows permanent water, light blue indicates seasonal water, and purple represents no water. The Occurrence Change Intensity layer (Fig. 17b) compares surface water between two epochs (1984–1999 and 2000–2021), using homologous months for consistent assessment. Here, red indicates water loss, green shows an increase, blue reflects no change, and brown marks no water. The Seasonality map (Fig. 17c) for the year 2021 shows intra-annual water presence, where blue represents permanent water, green seasonal water, and purple no water. The Recurrence map (Fig. 17d) depicts inter-annual return frequency of water, with blue showing permanent recurrence, green indicating seasonal recurrence, and purple areas of no recurrence. The Transitions layer (Fig. 17e) presents changes in water seasonality between the first and last representative observation years, showing different categories in distinct colors: no water, permanent water, seasonal, new seasonal, lost seasonal, seasonal to permanent, permanent to seasonal, ephemeral permanent, and ephemeral seasonal. Lastly, the Maximum Water Extent map (Fig. 17f) displays all locations where water was ever detected across the 38 years, marked in a range of colors to indicate maximum spatial coverage. Collectively, these six spatial layers provide essential information for evaluating wetland stability, transformation, and habitat availability factors that are crucial for understanding and conserving crane populations and their seasonal movements.

#### Role of land surface flow and water bodies in crane habitat and migration patterns

**Fig. 18:**
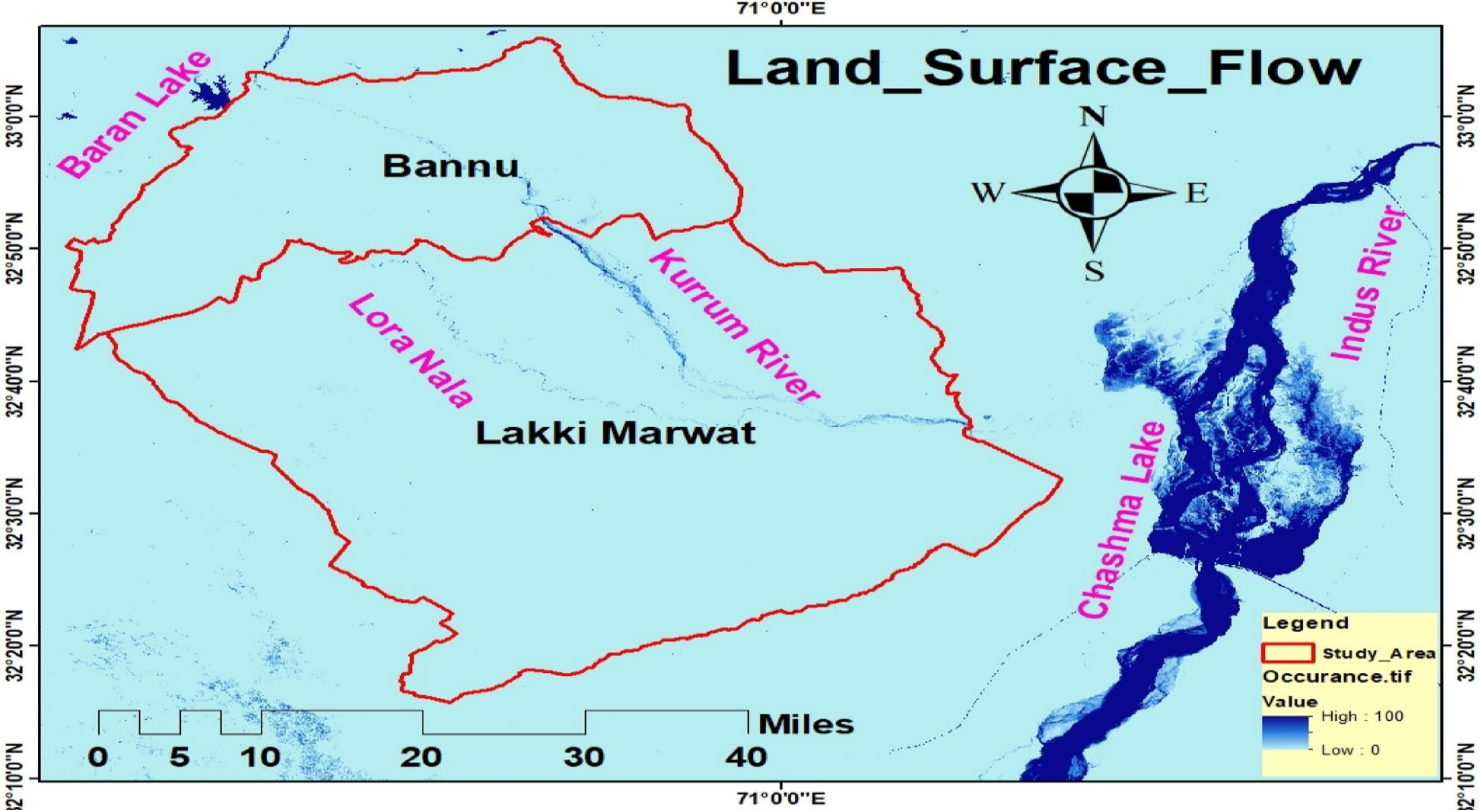
Land surface flow and water bodies.

In this study, LSF was also derived from the JRC Global Surface Water dataset using GIS techniques. The LSF map of the study area (Fig. 18) highlights key flyways for cranes, particularly along the Kurram River and Lora Nala. On one side of the study area lies Baran Lake, while on the other side are two important water bodies Indus River and Chashma Lake, both of which serve as critical habitats and stopover sites for migratory cranes. However, increasing land use/land cover (LULC) changes, population growth, resource exploitation, industrial expansion, pollution, global warming, and the broader impacts of climate change are having a significant negative effect on crane habitats, altering their traditional migration routes and timing, and disrupting established migratory patterns that are crucial for their survival.

## Discussions

Conserving migrating crane populations requires an understanding of habitat dynamics, particularly in areas where anthropogenic land use change, habitat fragmentation, and climate variability are putting increasing pressure on the species^73–75^. Our multi-index and long-term remote sensing analysis reveals significant shifts in habitat suitability for Demoiselle and Eurasian cranes in Pakistan over the past three decades. Our LULC changes from 1994 to 2024, including a sharp increase in built-up areas (+ 22.4%) and a notable decrease in vegetation cover (–4.2%), indicating intensifying habitat fragmentation and ecological stress. Our study aligns with similar global research on LULC and environmental changes and their effects on crane habitat, population, and distribution^19,20,25,72,73,75–77^. These results are in good agreement with international research, such as the 40% recorded decrease in shorebird habitat quality in China’s Yellow River Delta between 1975 and 2020 as a result of expanding mariculture and land reclamation, which broke up once-continuous wetland habitats^78^. Similarly, there have been reports of habitat loss in Estonia’s wetland systems and Ethiopia’s Lake Tana Biosphere Reserve due to hydrological changes and agricultural encroachment^19,79^. Like these international cases, our results show a reduction in surface water extent (via NDWI and MNDWI), vegetation cover fluctuation (NDVI, LSWI), and increased pressure on key seasonal foraging grounds, particularly during autumn and winter. The availability, spatial distribution, and continuity of suitable sites are all being impacted by these habitat changes, indicating an increasing discrepancy between crane habitat preferences and the quickly changing landscape^70^. Seasonal preferences and larger ecological patterns, food availability, and predator avoidance influence migratory birds’ behavior^76,80^. According to various studies^73,81–84^, Crane species have a significant degree of flexibility in modifying their flight path and migration pattern. Moreover, our spatial-temporal analysis revealed a decline in habitat quality and continuity over time, especially post-2010. This is consistent with research from the Yellow River Delta, which shows that between 1975 and 2020, the connectivity of suitable habitat decreased by over 60%, mostly as a result of increased fragmentation and changes in land use^85^. In Pakistan, the rapid expansion of agriculture, settlements, and road networks in wetland-adjacent areas contributed to increased edge effects, reduced patch size, and loss of key roosting and foraging grounds. Declining water levels and drought events also played a role in reducing surface water availability, as detected by long-term NDWI and MNDWI trends. Globally, loss of wetland connectivity has been linked to population declines in migratory waterbirds along major flyways, including the East Asian-Australasian and African-Eurasian routes^86,87^. A similar finding was also suggested by another study^87–90^. Our research contributes to this expanding body of evidence by showing that migrating cranes are becoming more and more confined to solitary habitat areas, which increases competition and lowers breeding success. As documented in studies of red-crowned and Demoiselle cranes in other parts of Asia, we saw a spatial concentration of favorable habitats in the winter, which may have resulted from migratory influxes from other regions that boosted territorial behavior^91,92^. During this investigation, it was noted that the majority of migrating crane species passed through the Kurram and Kashoo rivers. According to^77^ and Rehman, Alam^77^, numerous crane species have been reported in the vicinity of Kurram and the Kashoo River. Additionally, our study discovered a notable rise in settlements, a sign of population expansion, which ultimately affects crane habitats by contributing to increased pollution levels. Rehman, Alam^77^ found that pollution in the Kurram and Kashoo Rivers posed a serious threat to the Demoiselle crane. According to a different study, untreated sewage from homes and businesses severely pollutes the environment around cranes and their travel paths^92^. In particular, controlling the growth of weeds, replanting native plants like cypress, and controlling water levels in important wetlands helps meet the needs for shelter and food^93^. Furthermore, conservation planning should place a high priority on habitat connectivity to guarantee that cranes can travel freely across landscapes without encountering major human obstacles^95^. Lastly, although our research used long-term datasets and sophisticated remote sensing indices, future studies would benefit from incorporating field-based telemetry data and species-specific foraging behaviour to more accurately calibrate habitat suitability models^96^. Long-term changes to habitats identified through datasets including Land Use and Land Cover data (LULC), Normalized Difference Vegetation Index data (NDVI), and various water indices highlight degradation and fragmentation that greatly impact migratory crane species. Habitat restoration efforts, which include wetland restoration and afforestation, should be in locations where either water or vegetation trends have declined in order to improve foraging, roosting, and habitat connectivity for cranes. Areas used as stopover sites by cranes can be protected through habitat zoning and prohibitions of urban and agricultural development in the vicinity. By combining remote sensing and field-based monitoring, priority conservation actions and improvements for Demoiselle and Eurasian cranes can be identified to achieve long-term sustainability. In addition to improving model accuracy, this would match conservation initiatives with the real ecological needs of migrating crane populations. These findings make the necessity of strategic habitat protection very evident. GIS and remote sensing (RS) play a vital role in analyzing environmental changes and their effects on migratory birds. Globally, numerous comprehensive studies have been conducted in this domain. Na, Zang^96^ conducted a detail study on assessing breeding habitat suitability for the endangered red-crowned crane (Grus japonensis) based on multi-source remote sensing data. Another similar study conducted on Land use land cover change and public perceptions differently affect black crowned crane (Balearica pavonina) conservation: Evidences from Jimma zone southwestern Ethiopia^20^. Similar comprehensive study conducted on simulation of Spatial and Temporal Patterns of Suitable Wintering habitats for the Hooded Crane (Grus monacha) Under Climate and Land Use Change Scenarios by Jiang, Shao^78^. In the coastal area of northern Jiangsu Province, China, a study was carried out on the Effects of land-use change on the distribution of the wintering red-crowned crane (Grus japonensis)^97^. Spatio-Temporal Distribution Patterns and Determinant Factors of Wintering Hooded Cranes (Grus monacha) Population study carried out by Xu, Dong^24^. Our study shows that environmental changes, especially LULC shifts, significantly impact crane habitats. Continued habitat loss may threaten their survival, highlighting the need for stronger conservation measures at the policy level. This study has several limitations that should be considered when interpreting the results. First, the spatial resolution of Landsat imagery may not capture fine-scale habitat features, such as small wetlands or scattered vegetation patches, potentially underestimating crane habitat availability. Second, temporal uncertainties in NDVI, NDWI, MNDWI, and LSWI analyses may arise from seasonal data gaps, cloud cover, or sensor inconsistencies, which could affect the accuracy of long-term trends. Third, the absence of field-based crane occurrence, telemetry, or species-specific foraging data limits the ability to directly validate habitat suitability and infer actual responses of cranes to environmental changes. Additionally, inherent classification errors in LULC mapping and uncertainties in remote sensing indices may influence the precision of detected landscape changes. These limitations underscore the need for future studies to integrate higher-resolution imagery, comprehensive field surveys, and multi-source validation to more accurately assess habitat dynamics and guide conservation strategies for migratory cranes.

## Conclusion and recommendations

This study integrates land use/land cover data, three decades of climate records, and remote sensing indices (NDVI, NDWI, MNDWI, and LSWI) to give a geospatial assessment of habitat conditions for Demoiselle and Eurasian cranes in Pakistan’s Bannu and Lakki Marwat districts. The analysis identifies significant changes in land cover between 1994 and 2024, such as a steady decrease in barren land and an approximate 22.4% rise in built-up areas and a 4.2% decrease in vegetation cover. These trends imply increased human influence on natural landscapes, which may contribute to habitat fragmentation. Wetland-dependent species may benefit from the minor improvements in surface water availability shown by water-related indices, while vegetation indicators revealed temporal changes in vegetation health. Wetland dynamics and the timing of crane migration may be impacted by the little increase in average temperature (0.9 °C) and higher variability in monsoonal rainfall found in the climate assessment. The Kurram River and Lora Nala appear to serve as major migration paths for crane species; nevertheless, these habitats may be increasingly influenced by population increase, land change, and environmental constraints. While the findings offer helpful insights into long-term habitat changes, the study is hindered by the spatial resolution of satellite data and insufficient field-based validation, which should be addressed in future work. Overall, the findings highlight the need for more effective habitat management techniques, such as safeguarding and restoring important wetlands and water bodies, controlling unplanned urban growth, encouraging afforestation, and stepping up efforts to conserve animals. Incorporating remote sensing technology into long-term monitoring, together with community involvement and climate-informed conservation planning, may enhance the sustainability of crane populations and assist in maintaining the biological integrity of these migratory corridors.

## Data Availability

All data supporting the findings of this study are available within the paper. The data supporting the findings of this study are available from the corresponding author (WU Qingming) upon reasonable request.
